# Changes in Skeletal Muscle PAK1 Levels Regulate Tissue Crosstalk to Impact Whole Body Glucose Homeostasis

**DOI:** 10.3389/fendo.2022.821849

**Published:** 2022-02-11

**Authors:** Karla E. Merz, Ragadeepthi Tunduguru, Miwon Ahn, Vishal A. Salunkhe, Rajakrishnan Veluthakal, Jinhee Hwang, Supriyo Bhattacharya, Erika M. McCown, Pablo A. Garcia, Chunxue Zhou, Eunjin Oh, Stephanie M. Yoder, Jeffrey S. Elmendorf, Debbie C. Thurmond

**Affiliations:** ^1^ Department of Molecular & Cellular Endocrinology, Arthur Riggs Diabetes and Metabolism Research Institute of City of Hope, Duarte, CA, United States; ^2^ Department of Diabetes Complications and Metabolism, Arthur Riggs Diabetes and Metabolism Research Institute of City of Hope, Duarte, CA, United States; ^3^ Sahlgrenska Academy, Institute of Neuroscience and Physiology, Metabolism Research Unit, University of Gothenburg, Gothenburg, Sweden; ^4^ Division of Translational Bioinformatics, City of Hope, Duarte, CA, United States; ^5^ Global Scientific Communications, Eli Lilly & Company, Indianapolis, IN, United States; ^6^ Department of Anatomy, Cell Biology and Physiology, Center for Diabetes and Metabolic Disease, Indiana University School of Medicine, Indianapolis, IN, United States

**Keywords:** diabetes, insulin resistance, skeletal muscle, crosstalk, PAK1

## Abstract

Skeletal muscle accounts for ~80% of insulin-stimulated glucose uptake. The Group I p21–activated kinase 1 (PAK1) is required for the non-canonical insulin-stimulated GLUT4 vesicle translocation in skeletal muscle cells. We found that the abundances of PAK1 protein and its downstream effector in muscle, ARPC1B, are significantly reduced in the skeletal muscle of humans with type 2 diabetes, compared to the non-diabetic controls, making skeletal muscle PAK1 a candidate regulator of glucose homeostasis. Although whole-body PAK1 knockout mice exhibit glucose intolerance and are insulin resistant, the contribution of skeletal muscle PAK1 in particular was unknown. As such, we developed inducible skeletal muscle-specific PAK1 knockout (skmPAK1-iKO) and overexpression (skmPAK1-iOE) mouse models to evaluate the role of PAK1 in skeletal muscle insulin sensitivity and glucose homeostasis. Using intraperitoneal glucose tolerance and insulin tolerance testing, we found that skeletal muscle PAK1 is required for maintaining whole body glucose homeostasis. Moreover, PAK1 enrichment in GLUT4-myc-L6 myoblasts preserves normal insulin-stimulated GLUT4 translocation under insulin resistance conditions. Unexpectedly, skmPAK1-iKO also showed aberrant plasma insulin levels following a glucose challenge. By applying conditioned media from PAK1-enriched myotubes or myoblasts to β-cells in culture, we established that a muscle-derived circulating factor(s) could enhance β-cell function. Taken together, these data suggest that PAK1 levels in the skeletal muscle can regulate not only skeletal muscle insulin sensitivity, but can also engage in tissue crosstalk with pancreatic β-cells, unveiling a new molecular mechanism by which PAK1 regulates whole-body glucose homeostasis.

## Introduction

Currently 88 million people aged 18 years or older have prediabetes (34.5% of the US adult population) and 43.2 million (10.5% of the US population) have diabetes; half of prediabetic individuals develop type 2 diabetes (T2D) within 5 years (1). Prediabetes is characterized by elevated fasting blood glucose levels, ranging from 100 to 125 mg/dl, and/or impaired glucose tolerance. Prediabetes can be reversed and type 2 diabetes (T2D) averted, making prediabetes a compelling target for therapeutic intervention. However, current treatments are largely inadequate, limited to lifestyle modifications (diet, exercise) which can be unattainable or unsustainable over time. Therefore, there is an urgent need to develop new therapeutic agents that can reverse prediabetes and maintain normoglycemia (1).

Prediabetes is characterized by peripheral insulin resistance, such that the peripheral tissues like skeletal muscle and adipose tissue fail to appropriately respond to the insulin released from the pancreas. Without the ability to respond to the cue from insulin, the peripheral tissues do not launch the necessary cascade of events required to clear circulating glucose, ultimately leaving elevated concentrations of glucose in circulation. The primary regulator of glucose uptake in the body is the skeletal muscle, which is responsible for over 80% of glucose clearance (2). Insulin-stimulated GLUT4 translocation is a well-established mechanism by which insulin drives glucose transport into muscle (3, 4). The translocation of GLUT4 vesicles from intracellular sites to the PM occurs in response to insulin stimulation *via* the canonical insulin signaling pathway, whereby insulin binding to its membrane receptor activates phosphatidylinositol 3-kinase (PI3K) signaling. This signaling mechanism can use the well-described AKT-AS160 pathway or an alternative pathway that involves the Ras-related C3 botulinum toxin substrate1 (Rac1) (5). Rac1 is a Rho GTPase that regulates insulin-stimulated GLUT4 translocation to the PM by modulating actin cytoskeletal remodeling (5). Rac1 stimulates cytoskeletal remodeling *via* p21-activated kinase (PAK1), a serine threonine kinase with a GTPase binding domain (CRIB domain). The PAK1 homodimer becomes autophosphorylated and activated when Rac1 binds to the CRIB domain (6).

We and others have shown that PAK1 in skeletal muscle cells *in vitro* can modulate filamentous (F)-actin remodeling by regulating cofilin and the ARPC1B subunit of the ARP2/3 complex (7–9). *In vivo*, the absence of PAK1 in classic whole body knockout mice impairs skeletal muscle GLUT4 translocation (10). Multiple groups have demonstrated these whole-body knockout mice to have impaired glucose homeostasis, although to differing degrees (10–12). While reduced human skeletal muscle PAK1 and Rac1 activation levels are associated with T2D and obesity (13), these are correlations that require testing to ascertain whether skeletal muscle-specific depletion of PAK1 is sufficient to impair glucose homeostasis by dampening GLUT4 translocation.

Towards this, in this study, we describe key findings garnered from skeletal muscle-specific PAK1 knockout mice (skmPAK1-iKO), as well as a new skeletal muscle-specific PAK1 enrichment mouse model (skmPAK1-iOE), which point to the importance of skeletal muscle PAK1 in maintaining glucose homeostasis and identify crosstalk between skeletal muscle and pancreatic islet β-cells.

## Materials and Methods

### Human Skeletal Muscle

Cadaverous skeletal muscle samples from the leg muscles of 15 non-diabetic or type 2 diabetic donors were purchased from the National Disease Research Interchange (NDRI, Philadelphia, PA). The donors were Caucasian men and women between 45 and 78 years old (**Supplemental Table 1**). The procurement of human skeletal muscle biopsies from NDRI was approved by the Institutional Review Board at the University of Pennsylvania. The participants gave informed consent. The investigation was carried out in accordance with the principles of the Declaration of Helsinki as revised in 2008. The samples were snap-frozen and kept at -80°C until mRNA and protein extraction were carried out. mRNA was quantified from human skeletal muscle are previously described (14). Primers used for the detection of *hPAK1* (forward: 5’-GGAGTTTACGGGAATGCCAGAG-3’ and reverse: 5’-CAGCCTGCGGGTTTTTCTTC-3’); *hHPRT* (forward: 5’- TATGGCGACCCGCAGCCCT-3’ and reverse: 5’- CATCTCGAGCAAGACGTTCAG-3’) and *hARPC1B* (forward 5’-GCTGAGAGTACAGGTGCG-3’ and reverse 5’-CCTGCTGTGACCACACAC-3’).

### Immunoblot Analysis

Skeletal muscle was lysed using NP-40 lysis buffer as previously described (14). The samples were rotated at 4°C for 5 min and centrifuged at 17,100×g for 5 min. The supernatant was collected and aliquoted into tubes to avoid freeze/thaw protein degradation. Protein samples were denatured at 95°C for 5 min in freshly made Laemmli sample buffer containing dithiothreitol and β-mercaptoethanol) and proteins resolved on hand-casted 10% SDS-PAGE gels, using, and transferred onto PVDF membrane. The membranes were immunoblotted for PAK1 (Cell Signaling Technology #2602S, Danvers, MA,1:1000), ARPC1B (ECM Biosciences #ap4321, Versailles, KY,1:1000), TnFR (Invitrogen, #13-6800, Carlsbad, CA, 1:1000), GLUT4 (Santa Cruz Biotechnology, #sc-1608, Dallas, TX, 1:500), RhoGDI (Santa Cruz Biotechnology, #sc-360, 1:1000), STX4 (custom synthesized as described (15), 1: 2000), myc (Santa Cruz Biotechnology, #sc-40) or HPRT (Abcam #ab97698, Waltham, MA, 1:1000) in TBST + 1% BSA. For the secondary antibody, Goat Anti-Rabbit IgG (H L)-HRP Conjugate (Bio-Rad #172-1019, Hercules, CA), goat Anti-Mouse IgG (H L)-HRP Conjugate (Bio-Rad #172-1011) or donkey anti-goat IgG-HRP was incubated with the blot for 1 h at room temperature. Chemiluminescence was documented using a Bio-Rad ChemiDoc Touch and ECL (Amersham ECL Western Blotting detection reagent, GE Healthcare #RPN2106, Chicago, IL).

### Transgenic and Knockout Mice

All animal experiments were conducted in accordance with the NIH Guide for the Care and Use of Laboratory Animals (National Institutes of Health Publication no. 85-23, revised 1996) and approved by the Institutional Animal Care and Use Committees of City of Hope National Medical Center (Duarte, CA, USA; approval #15023). Mice carrying the tetracycline-response element (TRE)-hPAK1 transgene were generated on the C57BL6J background. Mice carrying the modified muscle creatine kinase (Mck)-reverse tetracycline-controlled transactivator (rtTA) construct were purchased from the Mutant mouse resource and research centers (MMRRC, cat. # 000422-MU) and maintained on the C57BL6J background. Custom generated heterozygous TRE-hPAK1^+/-^ mice have been crossed with heterozygous Mck-rtTA^+/-^ to generate skmPAK1-iOE mice.

Mice containing LoxP sites flanking the PAK1 gene **(**PAK1fl/fl) were obtained from Dr. Xin Wang (University of Manchester, UK) on a mixed background, and were backcrossed onto a pure C57BL6J background for >9 generations prior to crossing with human skeletal actin (HSA)-rtTA/TRE-Cre recombinase positive mice (Jackson Laboratory, # 012433, Bar Harbor, ME) to produce doxycycline-inducible on a pure C57BL6J background, to generate skmPAK1-iKO mice.

SkmPAK1-iKO mice were generated by providing 2 mg/ml doxycycline (Dox) in drinking water for 10 days. SkmPAK1-iOE mice were provided Dox in water starting at 12 weeks of age through the end of the experiments as described in the figure legends. Mice described as controls (CTRL) for skmPAK1-iKO experiments were PAK1fl/fl;HSA-rtTA/TRE-Cre mice with no Dox induction. Mice referred to as CTRL for skmPAK1-iOE consist of non-Dox-induced TRE-hPAK1^+/-^;Mck-rtTA^+/-^ mice or Dox-induced C57BL6J wild-type (WT) or Mck-rtTA^+/-^ mice. The C57BL6J background was chosen to enhance the detection of insulin resistance in the skmPAK1-iKO mice. Because insulin resistance can be detected by IPITT in male mice on the C57BL6J background whereas female mice are protected from peripheral insulin resistance (16), male mice were used for the skmPAK1-iKO studies. All experiments were repeated with at least 2 independent cohorts of mice with littermate mice used as controls within each cohort. All mice used in data depicted in IPGTT and IPITT studies are assessed as individual entities (e.g. the mean ± SEM of each mouse group represents raw data of each mouse of the n, non-normalized for cohort). Mice used for *in vivo* GLUT4 translocation data were grouped into three cohorts over the years of the study; data were normalized per cohort set to the basal control mouse; the other 3 mice of each set were normalized thereto (n=3, i.e. 4 animals/group and total of 12 animals). Mice were housed on Sani-Chip bedding in groups of 3-5 and fed standard maintenance chow (13% of kcal from fat; PicoLab #5053, Fort Worth, TX) starting at 4 weeks of age. Mice were obtained from heterozygous matings; each transgene in the iOE mouse model was maintained as heterozygous (e.g., TRE-PAK1^-/+^).

### GLUT4 Translocation to the Sarcolemmal/t-Tubule Membranes

The subcellular fractionation of skeletal muscle was performed as previously described (7, 14). Briefly, mice were fasted overnight, and then given an intraperitoneal injection of 21 U of Humulin R per kg of body weight (Eli Lilly & Co., Indianapolis, IN), or a similar volume of saline as a vehicle control. After 30 min, mice were assessed for blood glucose levels and then given an additional insulin injection. Mice were euthanized within 10 min and whole hindlimb muscles were rapidly collected for immediate homogenization in ice-cold homogenization buffer (20 mM Hepes, pH 7.4, 250 mM sucrose, 1 mM EDTA, 1 mM phenylmethylsulfonyl fluoride, 10 μg/ml aprotinin, 1 μg/ml pepstatin, 5 μg/ml leupeptin, and 5 mM benzamidine) using a Polytron PT-10 homogenizer, 3 times in 10-sec bursts. Samples were centrifuged at 2,000×g for 5 min at 4°C, and supernatant collected were then centrifuged at 9000xg for 20 min at 4°C. That supernatant from the 9000xg spin was subsequently centrifuged at 180,000xg for 90 min. Pellets containing the t-tubule and sarcolemmal membrane fractions were solubilized in detergent-containing buffer and proteins (~5 μg protein per lane). Proteins resolved by 10% SDS-PAGE for immunoblotting for GLUT4. GLUT4 band density was normalized to the scanned optical density of the Ponceau S stained full-length gel lane, to account for differential gel loading.

### Intraperitoneal Glucose Tolerance and Insulin Tolerance Testing

The intraperitoneal glucose tolerance test (IPGTT) was conducted within 3-4 weeks after dox induction, the time required to attain stable transgenic protein expression. For the IPGTT, mice were fasted for 6 h prior to experimentation (time between 0800–1400) for skmPAK1-iOE mice and overnight (time between 1800-0900) for skmPAK1-iKO mice and housed individually. D-glucose (2 mg/kg body weight) was diluted in saline, filtered and injected intraperitoneally. Blood glucose levels were measured immediately before injection (0 min) and then at 15, 30, 60, 90 and 120 min after injection. After injection, blood (2 µl) was taken from the mouse tail and diluted with 5 µl saline, then loaded into cuvettes and measured immediately using a HemoCue 120706 Glucose 201 Analyzer. Blood taken before injection was directly loaded into the cuvettes without dilution. For the intraperitoneal insulin tolerance test (IPITT), mice were dox-induced, fasted similarly as described for the IPGTT, and were injected intraperitoneally with Humulin R (0.75 units/kg body weight), and blood glucose levels were measured at 15, 30, 45, 60 and 90 min after injection.

### Body Composition Analysis

Whole body composition (fat and lean tissues) was determined using quantitative magnetic resonance technology (EchoMRI 3-in-1; Echo Medical Systems, Houston, TX). Automatic tuning and calibration of the instrument parameters using canola oil maintained at room temperature (22°C) was performed daily. One mouse was placed in the analytical chamber, and the following parameters were measured by the EchoMRI™: lean mass, fat mass, free water, and total water.

### Plasma Insulin Analysis

Blood was collected from mice that were fasted for 16 h. Plasma was separated after centrifugation at 6000 RPM for 10 min, and used to assess insulin (Insulin RIA kit, Millipore #SRI-13K, Burlington, MA) according to the manufacturers’ instructions.

### Cell Culture

L6-GLUT4-myc myoblasts, which were free from mycoplasma contamination, were purchased from Kerafast (Boston, MA, USA, cat. # ESK202-FP) were grown in MEM-α medium supplemented with 10% FBS (vol./vol.) and 1% (vol./vol.) antibiotic–antimycotic solution (Thermo Fisher, Waltham, MA, USA). L6-GLUT4-myc myoblasts were differentiated into myotubes by seeding and incubation in MEM-α medium containing 2% fetal bovine serum and 1% antibiotic-antimycotic solution. For insulin stimulation, the cells were fasted in serum free media for 50 min and then insulin stimulated (100 nM) for 10 min. The cells were harvested in 1% NP-40 lysis buffer containing 25 mM HEPES, pH 7.4, 1% Nonidet P-40, 10% glycerol, 50 mM sodium fluoride, 10 mM sodium pyrophosphate, 137 mM NaCl, 1 mM sodium vanadate, 1 mM phenylmethylsulfonyl fluoride, 10 µg/ml aprotinin, 1 µg/ml pepstatin, and 5 µg/ml leupeptin and cleared of insoluble material by centrifugation at 13,000×g for 10 min at 4°C. The supernatant was used for immunoblotting analyses. The cells were transfected with pCMV6-Myc-hPAK1 plasmid DNA using the Avalanche (EZ Biosystems LLC, Cat# EZT-L600, College Park, MD, USA) or K2 transfection reagents (Biontex Laboratories GmbH, Ca # T06, Munchen, Germany).

### *In Vitro* Cell Surface GLUT4-myc Detection

Cell surface levels of myc-tagged GLUT4 were detected using L6-GLUT4-myc myoblasts as previously described (7, 14, 17). In brief, L6-GLUT4-myc myoblasts were incubated in serum-free medium for 40 min, and then insulin added (100 nM) for 20 min, keeping cells at 37°C. Cells were then washed three times with ice-cold PBS, then fixed with 4% paraformaldehyde in PBS for 20 min at room temperature. Fixed (non-permeabilized) cells were then blocked in Odyssey blocking buffer (LI-COR Biosciences, Lincoln, NE, USA) for 1 h at room temperature, and incubated with mouse anti-Myc (1:100) overnight at 4°C. Cells were thoroughly washed and subsequently incubated with infrared-conjugated goat anti-mouse secondary antibody for 1 h at room temperature. Immunofluorescence intensity was quantified using the Odyssey CLx infrared imaging system (LI-COR Biosciences, Lincoln, NE). Data were normalized to SYTO60 (Invitrogen, Carlsbad, CA, USA), a red fluorescent nucleic acid stain.

### Adenoviral Transduction

N-terminal GFP tagged hPAK1 plasmid was generated in pCDNA3 (pCDNA3-GFP-hPAK1), and the GFP-hPAK1 insert subcloned into the pAd5-CMV adenoviral vector. Adenoviruses were generated and purified at Viraquest Inc. (North Liberty, IA). L6 myotubes were transduced at 500 MOI.

### Conditioned Media Assay

L6-Glut4-myc-myoblasts were transfected with pCMV6 vector (Ctrl) or pCMV6-hPAK1 plasmid DNAs for 48 h and media was harvested. Myotubes differentiated from L6-Glut4-myc-myoblasts, using 2% FBS containing MEM-α media for 8 days, were transduced with Ad-CMV-GFP or Ad-GFP-hPAK1 adenoviruses, and 48 h later conditioned media was harvested. Myoblast or myotube-derived conditioned media (CM) was mixed with RPMI (1:1 ratio) containing low glucose (2.5 mM) and low serum (2.5%) and incubated with INS-1 832/13 cells for 16 h. The INS-1 832/13 cells (provided by Dr. Christopher Newgard, Duke University Medical Center, Durham, NC) were subsequently washed and incubated in Krebs-ringer bicarbonate buffer (KRBB) for 1 h. Next, the cells were incubated in 1 mM or 20 mM glucose containing KRBB for 30 min and the supernatants were collected for determination of insulin released into the media using the rat insulin ELISA kit (ALPCO Cat#80-INSRT-E01, Salem, NH).

### RNA Isolation and RNA-Seq Analysis

RNA was isolated using the TriReagent according to the manufacturer’s protocol (MilliporeSigma, St. Louis, MO, USA). Reads were aligned against the mouse genome (mm10) using TopHat2 (18). Read counts were tabulated using htseq-count (19), with UCSC known gene annotations (TxDb.Mmusculus.UCSC.mm10.knownGene (20). Fold-change values were calculated from Fragments Per Kilobase per Million reads (FPKM) (21) normalized expression values, which were also used for visualization (following a log_2_ transformation). Aligned reads were counted using GenomicRanges (22). Two different p-value estimation methods were used to discover differentially expressed genes for the knock-out comparison [edgeR, (23)] and the over-expression comparison [DESeq2, (24)]. Prior to p-value calculation, genes were filtered to only include transcripts with an FPKM expression level of 0.1 (after a rounded log2-transformation) in at least 50% of samples (25) as well as genes that are greater than 150 bp. While the code has to be modified for every project, these scripts are a modified version of a template for RNA-Seq gene expression analysis (https://github.com/cwarden45/RNAseq_templates/tree/master/TopHat_Workflow). The raw and processed data is available in GSE191000. The following comparisons were run: a) Group 2 vs Group 3 (female skmPAK1-iOE mice vs. non-Dox-induced TRE-hPAK1^+/-^;Mck-rtTA^+/-^ as Control) and b) Group 4 vs. Group 5 (male skmPAK1-iKO mice vs. Control). All differential expression analyses were performed using R (26) and R packages readxl (27) and tidyverse (28). The heatmap and scatter diagrams were created using the packages ggplot2 (29), gplots (30) and ggrepel (31).

### GO Enrichment Analysis

By analyzing the gene-wise fold changes, we created the following gene sets: a) differentially expressed between iKO and wild type, b) upregulated in iKO, c) downregulated in iKO, d) differentially expressed in iOE over wild type, e) upregulated in iOE, f) downregulated in iOE, g) downregulated in iKO and upregulated in iOE. We performed the biological process enrichment analysis for each of these gene sets separately using the entire mouse transcriptome as the reference (universe). For each of the gene sets, a hypergeometric test was conducted and processes that are relevant to diabetes with a p value < 0.001 were reported. All analyses were performed using the R libraries GO.db (32) and GO stats (33).

### STRING Protein-Protein Interaction Network Analysis

From the GO analysis, we retrieved the genes that were downregulated in KO and upregulated in OE and enriched in diabetes related processes. These genes were used to search the STRING database for annotated protein-protein interactions. The subnetwork involving the query genes as well as the first and second shell of interactions were reported. The analysis was performed online at https://string-db.org (34). Following is the list of genes that were used for searching the STRING database: Scd1, Ptk2b, Lgals12, Pck1, Ptgs2, Adipoq, Pde3b, Bcl11b, Prkcb, Bcar3, Pak1, Aoc3, Ffar4.

### Statistics

All data are presented as mean ± SEM. Differences between two groups were assessed using Student’s t-test. Statistically significant differences among multiple groups were evaluated using one-way ANOVA followed by the *post-hoc* test indicated in the figure legends. Details of each statistical test are indicated in the figure legends. P<0.05 was the threshold for significance and all analysis were performed using GraphPad Prism software, version 9.

## Results

### PAK1 and ARPC1B Protein Levels Are Reduced in T2D Human Skeletal Muscle

**Figure 1:**
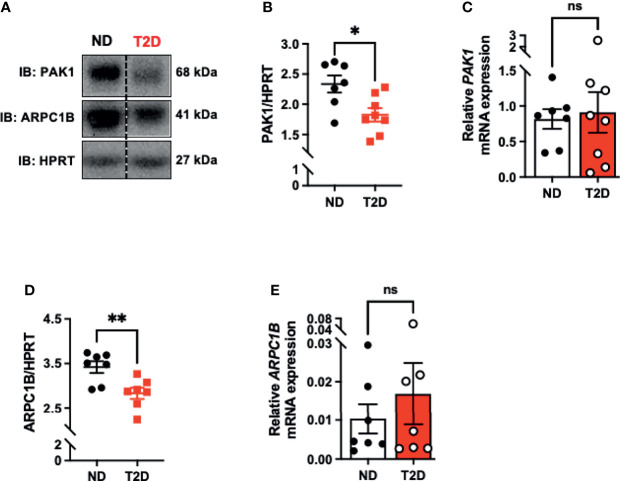
PAK1 protein abundance is decreased in type 2 diabetic human skeletal muscle. **(A)** Immunoblot analysis of cadaveric skeletal muscle tissue from type 2 diabetic (T2D) and non-diabetic (ND) individuals. **(B)** PAK1 protein abundance quantification in ND and T2D individuals; n=7-8 per group. **(C)** PAK1 mRNA expression normalized to HPRT in skeletal muscle RNA. T2D: n=8: ND: n=7. **(D)** ARPC1B protein abundance; n=7 per group. **(E)** ARPC1B relative mRNA expression (normalized to HPRT); n=7 per group. *p < 0.05, **p < 0.01, ns; not statistically significant, Two-tailed unpaired student’s t-test.

Assessment of PAK1 protein and mRNA levels in skeletal muscle from age- and gender-matched non-diabetic and T2D cadaveric donors (**Supplementary Table 1**) revealed a significant reduction in PAK1 protein abundance in T2D individuals relative to non-diabetic controls (**Figures 1A, B**). However, we found no significant reduction in *PAK1* mRNA levels in T2D individuals relative to non-diabetic controls (**Figure 1C**). PAK1 protein abundance was not associated with age or body mass index of the donor (**Supplementary Figures 1A, B**). We also evaluated levels of ARPC1B, a key PAK1 effector in skeletal muscle, which regulates GLUT4 vesicle trafficking (7). Like PAK1, ARPC1B protein abundance was reduced in skeletal muscle of T2D donors relative to non-diabetic donors (**Figure 1D**), with mRNA levels not significantly different between the non-diabetic and T2D groups (**Figure 1E**). These data suggest that PAK1 and ARPC1B proteins are reduced at a post-transcriptional level, possibly in response to the T2D milieu. These findings are consistent with prior studies of these factors regulating GLUT4 vesicle trafficking in skeletal muscle cells (7, 35).

### Skeletal Muscle-Specific Deletion of PAK1 Impairs Insulin Sensitivity

**Figure 2:**
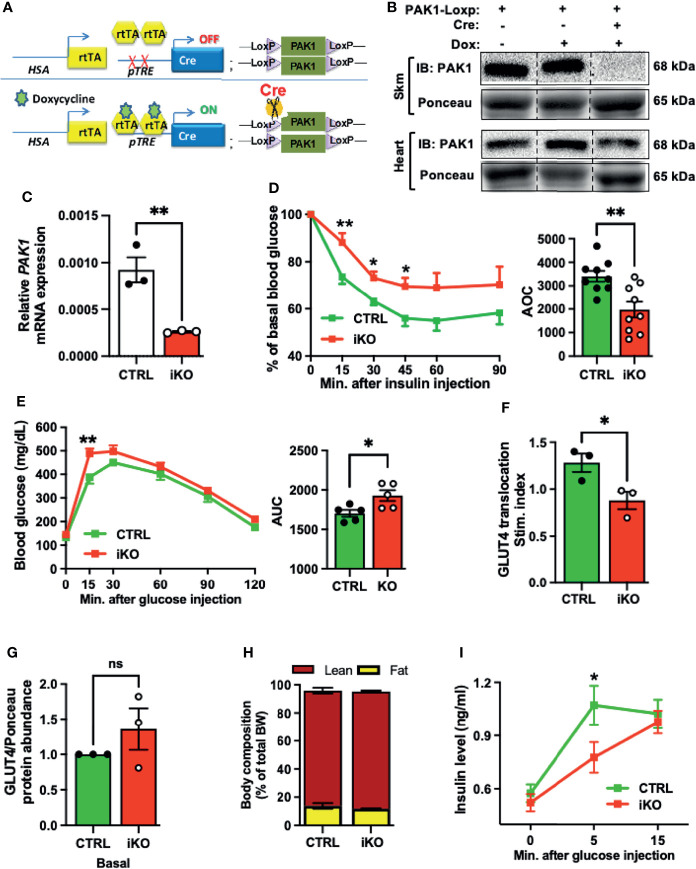
Skeletal muscle-specific PAK1 knockout leads to glucose intolerance and insulin resistance in male mice. **(A)** Gene schematic of the doxycycline (Dox)-inducible skeletal muscle-specific PAK1 knockout (skmPAK1-iKO, iKO) mouse model (HSA, human skeletal actin; rtTA, reverse tetracycline transactivator; pTRE, tet-response element). **(B)** Immunoblot analysis of skeletal muscle and heart tissue from control (lanes 1-2) and skmPAK1-iKO (lane 3) male mice. **(C)** mRNA expression of *Pak1* in mouse skeletal muscle of control (CTRL) and skmPAK1-iKO (iKO) mice, n=3 per group. **(D)** Intraperitoneal insulin tolerance test (IPITT) of CTRL (green) and iKO (red) male mice, n=9 per group, with quantification of the area over the curve (AOC). **(E)** Intraperitoneal glucose tolerance test (IPGTT) of CTRL and iKO mice with quantification of the area under the curve (AUC), n=5 per group. **(F)** *In vivo* insulin-stimulated GLUT4 translocation to the sarcolemmal/t-tubule membrane P2 fraction in mouse hindlimb, expressed as stimulation index (each normalized to un-induced muscle), n=3 per group. **(G)** GLUT4 protein abundance in P2 fractions of fasted (basal condition) CTRL and iKO mice, n=3 per group. **(H)** Body composition analysis for CTRL and iKO mice. BW, body weight. **(I)** Fasting plasma insulin levels before glucose injection (time 0), and at 5 and 15 minutes after glucose injection during the IPGTT in CTRL (green line) and iKO (red line) male mice; n=5-10 per group. *p < 0.05, **p < 0.01, ns: not statistically significant, two-tailed, unpaired student’s t-test (C-H, bar graphs). Unpaired two-sample t-test, Holm-Sidak’s multiple comparisons test [**(D, E, I)**, line graph].

To model the loss of PAK1 and its downstream effector in T2D muscle, and to evaluate the importance of skeletal muscle PAK1 in glucose homeostasis *in vivo*, we generated skmPAK1-iKO mice. Dox administration was used to deplete PAK1 *via* the following mechanism. The HSA promoter (36) drives skeletal muscle-specific expression of rtTA, which, in the presence of Dox, binds the TRE (37). This binding activates expression of Cre, which targets the LoxP sites bracketing the mouse *Pak1* gene, resulting in *Pak1* excision (**Figure 2A**). Skeletal muscle homogenates from Dox-induced mice carrying both the PAK1fl/fl and HSA-rtTA/TRE-Cre constructs showed selective loss of PAK1 protein (>95%), relative to controls carrying only PAK1fl/fl (**Figure 2B**), and showed significant reduction (>80%) in skeletal muscle *Pak1* mRNA levels (**Figure 2C**).

Next, we assessed whether skeletal muscle PAK1 regulates peripheral insulin sensitivity *in vivo*. As expected, the intraperitoneal insulin tolerance test (IPITT) revealed significant peripheral insulin resistance in the skmPAK1-iKO mice, with area over the curve (AOC) decreased by ~35% (**Figure 2D**); Dox was confirmed to be without effect on IPITT (**Supplementary Figure 2A**). Intraperitoneal glucose tolerance tests (IPGTT) revealed glucose intolerance in the skmPAK1-iKO mice (**Figure 2E**), with area under the curve (AUC) significantly increased compared to AUC of control (CTRL) mice. To determine if the peripheral insulin resistance in the skmPAK1-iKO mice was underpinned by defects in GLUT4 vesicle translocation, mice were injected with insulin, the whole hindlimb muscle was rapidly excised for subcellular fractionation, and levels of GLUT4 that had translocated into the sarcolemmal/t-tubule membranes (P2 fraction) were quantified. P2 fraction purity was validated by the presence of plasma membrane-localized proteins such as Transferrin Receptor (TnFR) and Syntaxin 4 (STX4) as described for this method (38, 39), and the absence of the cytosolic protein RhoGDI (**Supplementary Figure 2B**). Consistent with our hypothesis, the skmPAK1-iKO P2 fractions showed reduced insulin-stimulated GLUT4 accumulation in the sarcolemmal/t-tubule membranes, relative to unstimulated/basal levels, compared with that of CTRL (**Figure 2F**); basal levels of GLUT4 in the P2 fraction were not significantly different (**Figure 2G** and **Supplementary Figure 2C**), validating prior work from L6 myotubes and myoblasts that PAK1 signaling is required to mobilize GLUT4 vesicles (35). In addition, the skmPAK1-iKO mice had no abnormalities in body and tissue mass (**Supplemental Table 2**) or body composition (**Figure 2H**). Interestingly, the plasma insulin levels were significantly slower to rise in response to a glucose challenge in the skmPAK1-iKO mice than in the CTRL mice (**Figure 2I**). The fasting plasma insulin level (time 0) was not different between skmPAK1-iKO and CTRL mice, and skmPAK1-iKO insulin levels reached the same level as those of CTRL mice by 15 min. This result suggested a novel possibility that skeletal muscle PAK1 protein levels influence, in some integrative fashion, the rate of release of insulin from the pancreatic β-cells.

### PAK1 Enrichment in Mouse Skeletal Muscle Increases Glucose Tolerance and Preserves GLUT4 Translocation Under Insulin-Resistance Conditions

**Figure 3:**
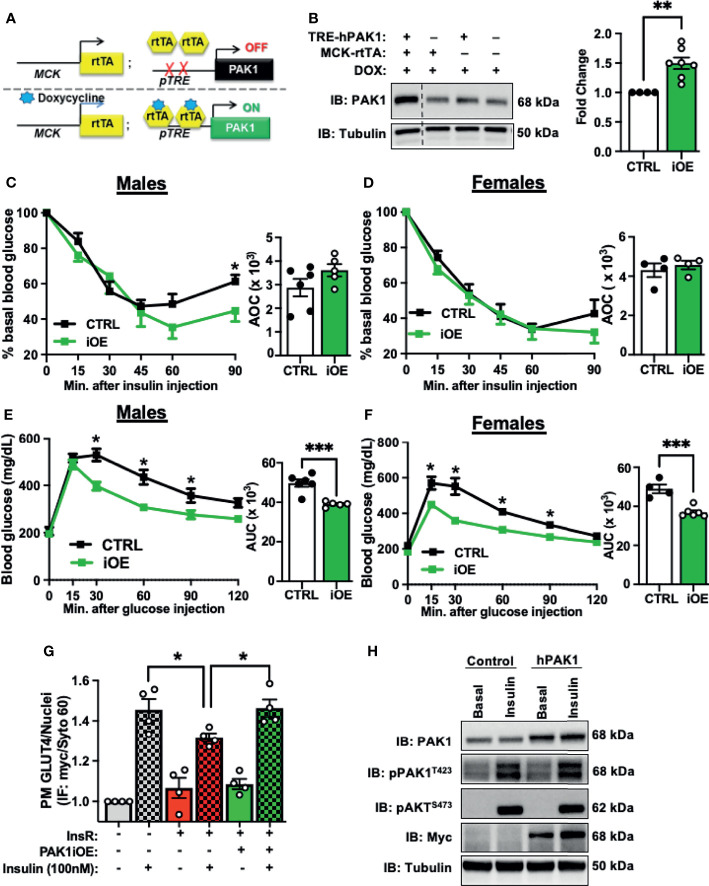
Skeletal muscle-specific PAK1 enrichment leads to improved glucose tolerance. **(A)** Gene schematic of the Dox-inducible skeletal muscle-specific PAK1 overexpression (skmPAK1-iOE) mouse model. **(B)** Immunoblot analysis of skeletal muscle from skmPAK1-iOE mice (lane 1) and control mice (lanes 2-4), with corresponding quantification (for each of 4 independent membranes containing one CTRL and multiple dTg+Dox samples, raw data were normalized to tubulin, CTRL set equal to 1, and all samples normalized thereto per membrane, to account for independent membranes), n=4-7 per group. **(C)** IPITT of control (CTRL, black) and skmPAK1-iOE (PAK1, green) male mice, with quantification of AOC. **(D)** IPITT of CTRL (black) and PAK1 (green) female mice, with quantification of AOC, n=4-5 per group. **(E)** IPGTT of CTRL and PAK1 male mice with quantification of AUC. **(F)** IPGTT of CTRL and PAK1 female mice with quantification of AUC, n=4-5 per group. **(G)** *In vitro* GLUT4 translocation in L6 myoblasts, expressed as plasma membrane GLUT4 puncta normalized to number of nuclei. Cells were treated with chronic insulin (5 nM for 12 h) followed by acute (100 nM) insulin stimulation. Grey bars (1–2) represent control cells with (+) and without (-) insulin stimulation. Red bars (3–4) show control cells exposed to insulin resistance (InsR) conditions with and without insulin stimulation, and green bars (5–6) show PAK1-enriched cells exposed to InsR conditions. **(H)** Resultant lysates were assessed for PAK1 content, phosphorylated PAK1 and pAKT by immunoblot.; n=4. *p < 0.05, **p < 0.01, ***p < 0.005, by two-tailed unpaired student’s t-test [**(B–F)** bar graph] Unpaired two-sample t-test, Holm-Sidak’s multiple comparisons test [**(C–F)**, line graph]. One-way ANOVA with Tukey *post-hoc* analysis **(G)**.

Given that PAK1 depletion in mouse skeletal muscle leads to insulin resistance and glucose intolerance, we sought to examine if PAK1 enrichment would promote insulin sensitivity and enhance glucose tolerance. We generated a skeletal muscle-specific transgenic mouse to model PAK1 enrichment in skeletal muscle (skmPAK1-iOE). Mice containing the human PAK1 gene (hPAK1) under control of the TRE promoter were generated and crossed with mice expressing rtTA under control of a muscle creatine kinase (Mck) promoter that was modified to not express in heart tissue (40) (**Figure 3A**). This resulted in selective Dox-inducible hPAK1 overexpression in skeletal muscle in adult skmPAK1-iOE mice (**Figure 3B**); PAK1 overexpression was not detected in tissues such as heart or liver (**Supplementary Figures 3A, C, D**). Male mice showed significantly improved sustained peripheral insulin-responsiveness at 90 min after insulin injection in the IPITT (**Figure 3C**). A similar trend was observed in females, although it did not reach statistical significance (**Figure 3D**). Similarly, the IPGTT revealed improved glucose tolerance in skmPAK1-iOE mice compared to CTRL in both male and female mice (**Figures 3E, F**, respectively).

Non-Dox induced mice carrying both transgenes (TREhPAK1;MCK-rtTA, no Dox) show ~2-fold less PAK1 than Dox-induced mice carrying both transgenes, and were not significantly different from non-Dox treated WT mice (**Supplementary Figure 3B**). In males and in females, non-Dox-induced TREhPAK1;MCK-rtTA mice, Dox-induced WT mice, and Dox-induced Mck-rtTA mice showed no differences in IPGTT and IPITT responsiveness (**Supplementary Figures 3G-H, J-K**). In addition, the skmPAK1-iOE mice had normal body and tissue mass (**Supplementary Tables 3-4** and **Supplementary Figures 3I, L**).

The small magnitude of the increase in insulin sensitivity conferred by skmPAK1-iOE in chow-fed normal adult mice suggests that skeletal muscle PAK1 levels are sufficient, and further increasing PAK1 levels does not lead to substantial gains in function. Therefore, we asked whether PAK1 enrichment can enhance function in models of T2D. Because PAK1 protein levels are reduced in skeletal muscle from T2D individuals, we used hPAK1-overexpressing L6-GLUT4-myc myoblasts exposed to chronic insulinemia (5 nM insulin for 12 h) as a model of insulin resistance (17). When GLUT4-myc vesicles translocate to and fuse with the PM, the myc-tag becomes exofacially accessible to detection by myc antibody under non-permeabilized cell conditions, serving as a readout of GLUT4 translocation to and integration into the PM. We hypothesized that PAK1 enrichment would protect against loss of insulin-stimulated GLUT4 translocation to the PM. As expected, insulin resistance treatment diminished the insulin-stimulated GLUT4-myc signal, denoting reduced GLUT4 translocation/PM integration, relative to control cells (**Figure 3G**, bar 4 vs. bar 2). Remarkably, when the L6 cells were transfected to overexpress human (h)PAK1, they resisted the dampening effects of the insulin resistance treatment on insulin-stimulated GLUT4 translocation to the PM (**Figure 3G**, bar 6 vs. bar 4). Myoblast transfection efficiency was ~50% (Supplementary **Figure 4A**), with myoblasts exhibiting normal insulin stimulated PAK1 activation (pPAK1^T423^) and pAKT^S473^ (**Figure 3H**), supporting the concept that the protective effect of PAK1 was downstream of AKT activation and at the level of GLUT4 translocation.

**Figure 4:**
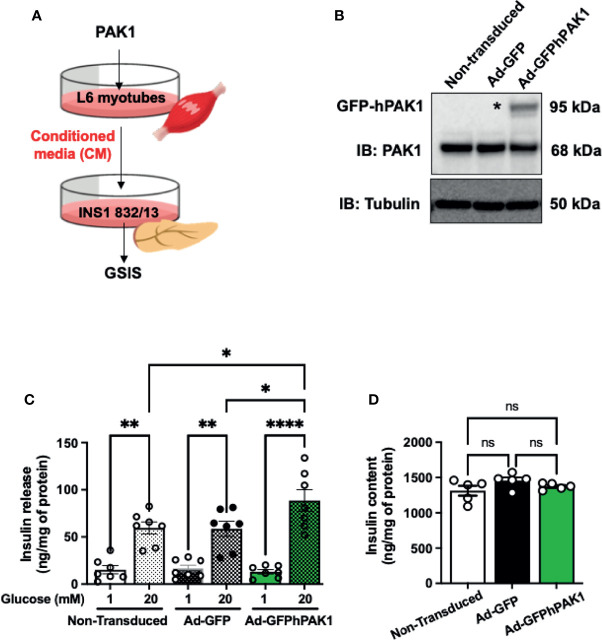
PAK1-overexpressing L6 myotube-derived conditioned media enhances GSIS in β-cells. **(A)** Schematic of the experimental design using PAK1-overexpressing myotubes. **(B)** Immunoblot demonstrating GFP-tagged hPAK1 overexpression in adenovirally transduced L6 myotubes (Ad-GFP-hPAK1) compared to non-transduced or vector control transduced (Ad-GFP) myotubes; image representative of n=5 independent passages of myotubes. **(C)** INS-1 832/13 cells were incubated with conditioned media (CM) from L6 myotubes in **(B)** for 16 h, media removed and replaced with MKRBB for assessment of insulin release, under low glucose (1 mM) conditions or high glucose (20 mM) for 30 min; n=7 per group. **(D)** Insulin content in INS-1 832/13 cells following GSIS analysis in **(C)**; n=5. One-way ANOVA with Tukey *post-hoc* analysis **(C, D)**, *p < 0.05, **p < 0.01, ****p < 0.0001. n.s., not significant.

### PAK1 in Skeletal Muscle Mediates Crosstalk Between Skeletal Muscle and β-Cells

The observation that skmPAK1-iKO reduces the rapid insulin response to glucose challenge (**Figure 2I**) led us to hypothesize that changes in skeletal muscle PAK1 levels might signal to the pancreatic β-cells. To test this hypothesis, we used a conditioned media (CM) paradigm wherein we applied media from L6 skeletal myotubes overexpressing hPAK1, to INS-1 832/13 clonal β-cells and measured changes in glucose stimulated insulin secretion (GSIS) from β-cells (**Figure 4A**). Adenoviral transduction of the myotubes was efficient (Supplementary **Figure 4B**) and yielded expression of the ~95 kDa GFP-tagged hPAK1 protein within 48 h (**Figure 4B**, denoted by asterisk*). The CM was collected and applied to INS-1 832/13 β-cells in culture for 15 h. In β-cells treated with the CM from Ad-GFP-hPAK1 myotubes, GSIS was significantly enhanced relative to that from control CM (non-transduced and GFP-transduced myotube CM) -treated β-cells (**Figure 4C**, bar 6 vs. bars 4 and 2). Basal levels of insulin release (measured in media containing 1 mM glucose, **Figure 4C**, bars 1, 3 and 5), and INS-1 832/13 clonal β-cell insulin content (**Figure 4D**), were not different between β-cells treated with control or GFP-hPAK1-enriched myotube-derived CM.

**Figure 5:**
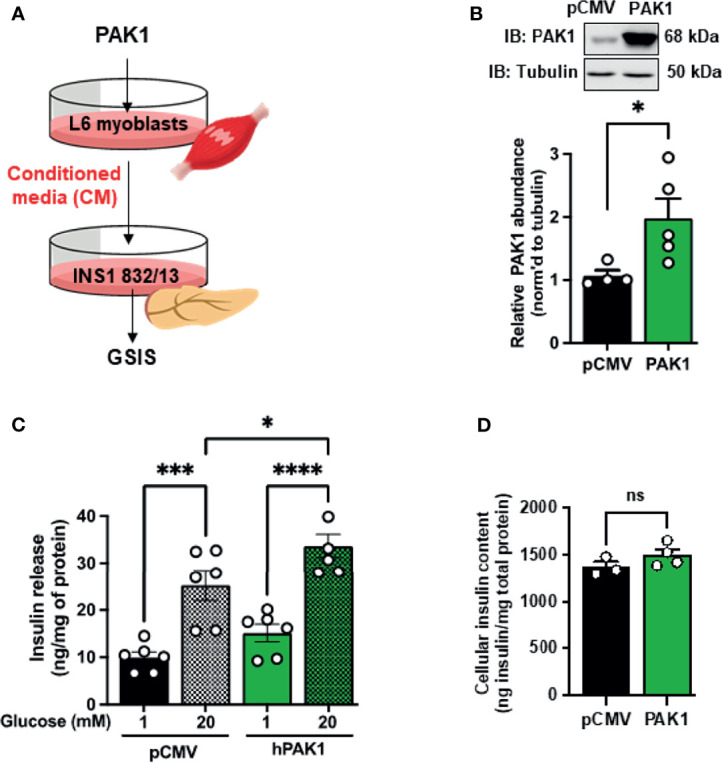
PAK1 overexpression in L6 myoblasts causes release of a factor(s) that promotes β-cell function. **(A)** Schematic of the experimental design. **(B)** Immunoblot demonstrating PAK1 overexpression in transiently-transfected L6 myoblasts (PAK1) compared to L6 myoblasts transfected with empty vector (pCMV), n=4-5. **(C)** Glucose-stimulated insulin secretion in INS-1 832/13 cells treated with conditioned media from PAK1 or pCMV transfected L6 myoblasts under low glucose (1 mM) conditions or high glucose (20 mM) for 30 min; n=5-6 experiments using independent passages of cells. **(D)** INS-1 832/13 cell insulin content; n=3-4 experiments using independent passages of cells. Two-tailed unpaired student’s t-test **(B, D)** or one-way ANOVA with Tukey *post-hoc* analysis **(C)**, *p < 0.05, ***p < 0.005, ****p < 0.0001. n.s., not significant.

In addition, this paradigm was also tested using L6 myoblasts, wherein CM collected from L6 myoblasts that were transiently transfected to overexpress PAK1 was applied to β-cells and GSIS assessed (**Figure 5A**). Transfected myoblasts expressed ~2-fold more PAK1 protein compared to endogenous PAK1 in the control pCMV-vector-transfected myoblasts (**Figure 5B**), and incubation of INS-1 832/13 β-cells with CM from the PAK1-overexpressing myoblasts resulted in enhanced GSIS relative to that of the β-cells incubated with CM from vector-expressing myoblasts (**Figure 5C**, bar 4 vs. bar 2); β-cell insulin content was unchanged (**Figure 5D**). The observation that CM from muscle cells overexpressing PAK1 enhances GSIS is unique to PAK1 overexpression to date, as other factors like Syntaxin 4 overexpressed (STX4 OE) in L6 myoblast cells had no enhancing effect upon GSIS compared with vector-transfected control cells (STX4 OE=11 ng insulin/mg total protein at 1 mM glucose; 24 ng insulin/mg total protein at 20 mM glucose, n=4, p > 0.05 vs. vector control (41). These data suggest that PAK1 levels in the skeletal muscle may mediate the release of factors into the circulation that crosstalk with the pancreatic β-cells.

### Identification of Candidate Genes Co-Associated With PAK1 in Skeletal Muscle

**Figure 6:**
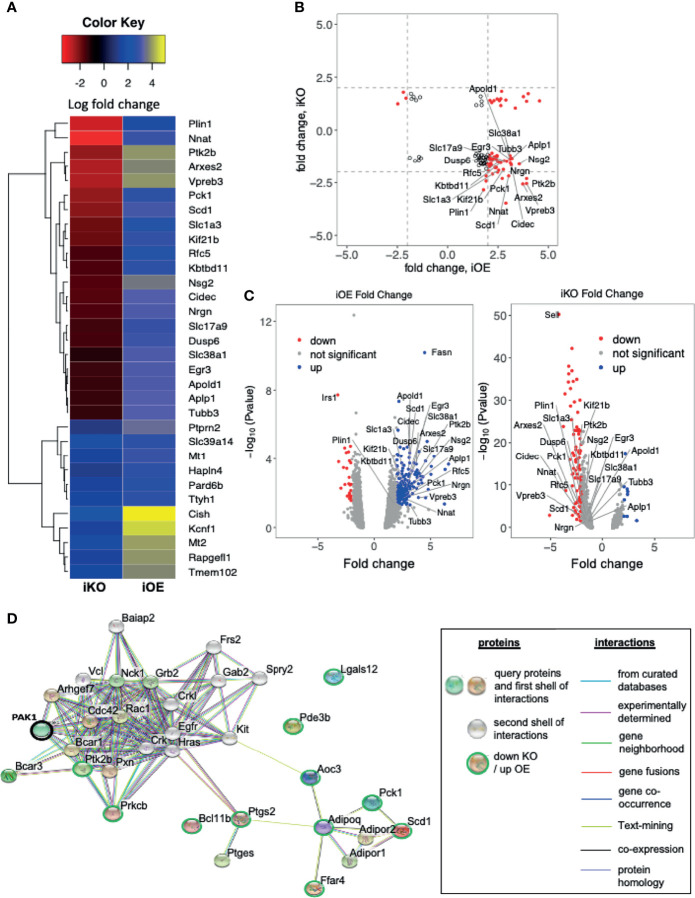
Changes in skeletal muscle PAK1 abundance led to modulation of genes: **(A)** Heatmap depicting the top genes that were differentially expressed (fold change > 2.5 or <-2.5, p < 0.05) in either skmPAK1-iKO vs. CTRL, or skmPAK1-iOE vs. CTRL skeletal muscle. The genes are colored according to the fold change in expression relative to the respective controls; the positive and negative signs represent up- and down-regulation, respectively. **(B)** Scatter diagram showing the fold change in gene expression for skmPAK1-iOE vs. CTRL, compared to skmPAK1-iKO vs. CTRL skeletal muscle (p<0.05); genes with a fold change >2 or <-2 are highlighted in red. **(C)** Volcano plots for genes in skmPAK1-iKO vs. CTRL skeletal muscle, or skmPAK1-iOE vs. CTRL skeletal muscle. Genes with p<0.05 and fold change >2 or <-2 are colored blue and red, respectively. In both B and C, genes that show a different direction of expression change between the two datasets are labeled. **(D)** The subnetwork was retrieved using the iKO down- vs iOE up- regulated genes from STRING database. PAK1 is circled in black, and the other genes are circled green.

To identify candidate genes co-associated with PAK1 in skeletal muscle, we conducted RNA sequencing analysis using skeletal muscles collected from mice used in the *in vivo* studies. Indeed, PAK1 is known to translocate to the nucleus where it can trigger alterations in the gene expression profile to exert impact on cellular function (42, 43). The list of altered genes was first filtered to include those with a fold change >2 or <-2 and p<0.05, followed by comparison of the skmPAK1-iKO and skmPAK1-iOE hits against each another. Genes on the candidate list for both skmPAK1-iKO and skmPAK1-iOE were identified for further focus (**Figure 6A**). Plotting the fold change of these genes for the skmPAK1-iOE vs. control comparison against the skmPAK1-iKO vs. control comparison revealed a cluster of genes that was upregulated with PAK1 enrichment and downregulated with PAK1 depletion (**Figure 6B**). Genes with expression levels that changed in the same direction for skmPAK1-iOE and skmPAK1-iKO were disregarded as being unrelated to the levels of PAK1 protein. The p-values (-log10[p value]) are shown relative to the fold change for the resulting candidate genes on volcano plots (**Figure 6C**). Gene expression levels found to be the most strongly associated with PAK1 expression levels, and overlapping between the skmPAK1-iKO and skmPAK1-iOE groups are (in order, beginning with the lowest p-values): *PLIN1, NNAT, PTK2B, ARXES2, VPREB3, PCK1, SCD1, SLC1A3, KIF21B, RFC5, KBTBD11, NSG2, CIDEC, NRGN, SLC17A9, DUSP6, SLC38A1, EGR3, APOLD1, APLP1, TUBB3* (**Supplementary Table 5**).

Gene ontology (GO) analyses were performed to better characterize the biological process enriched in skeletal muscle from skmPAK1-iKO and skmPAK1-iOE mice. The genes involved in negatively regulating apoptosis were significantly reduced in skmPAK1-iKO mice, in contrast to skmPAK1-iOE, where the negative regulators of apoptosis were significantly elevated (**Table 1**). The other biological processes, such as regulators of lipid metabolism, genes involved in response to insulin stimulus and glucose transmembrane transport, were all elevated in skmPAK1-iOE (**Table 1**), whereas downregulated in skmPAK1-iKO mice. Furthermore, differentially expressed genes identified by RNAseq from skmPAK1-iKO and skmPAK1-iOE mice were analyzed using the search tool for retrieval of interacting genes (STRING) database, which integrates known and predicted protein-protein interaction (PPI) networks. According to STRING analysis, there is a predicted protein-protein interaction between Adipoq (Adiponectin) and Ptgs2 (Prostaglandin-Endoperoxide Synthase 2, aka cyclooxygenase-2, COX-2) that may be under the regulation of PAK1 (**Figure 6D**).

**Table 1:** Gene-ontology (GO) analysis.

GOBPID	P value	Term	Condition	Genes
GO:0043066	1.34E-04	Negative regulation of apoptotic process	KO_down	Ptk2b, Egr3, Itprip, Ptgs2, Tcf7, Hcls1, Ffar4, Bcl11b, Cd27, Ntrk2, Ccnd2, Tnfaip3, Cbl, Madd, Mad2l1, C oro1a, Nckap1l, Fgf10, Cd74, Mdk, Lef1, Bmp7, Pik3cg, Pim1, Meis3, Cxcr2, Clcf1, Ccr7, Lep, Foxc2, Mmp9, Mag, Apoe, Cxcr3, Alox12, Dlx1, Tnfrsf4, Atad5, Jak3, Il7, Fcmr, Flt3l, Il7r, Fga, Brca1, Cd44, Rgn, Cx3cr1, Alb, Hck, Il2rb, Naip2, Rps6ka1, Naip5, Bcl3, Il18, Nuak2, Ccl5, Krt18, Fabp1, Plac8
GO:0043066	1.43E-07	Negative regulation of apoptotic process	OE_up	Plk3, Hnrnpk, Ptk2b, Egr3, Asns, Zc3h12a, Cx3cl1, Hspa5, Id1, Sgk1, Plk2, Rara, Prkcz, Dnajc5, Gabrb3, Pik3r1, Tyro3, Sfrp1, Itprip, Adar, Mertk, Hmgcr, Ptgs2, Mt1, Arl6ip1, Bmf, Mt3, Nr4a2, Stxbp1, Socs3, Dpep1, Tox3, Snca, Arnt2, Adora1, Prkcd, Pttg1ip, Camk1d, Pafah2, Mnt, Ung, Tcf7, Slc40a1, Hcls1, Gnaq, Eif2ak3, Ffar4, Gpx1, Faim2, Htt, Bcl11b, Mapk8ip2, Sphk1, Bmp4, Cttn, Plaur
GO:0019216	9.50E-05	Regulation of lipid metabolic process	KO_down/OE_up	Scd1, Ptk2b, Lgals12, Pck1, Ptgs2, Adipoq, Pde3b, Bcl11b
GO:0032869	1.46E-04	Cellular response to insulin stimulus	KO_down/OE_up	Prkcb, Pck1, Bcar3, Pak1, Adipoq, Pde3b
GO:0010827	4.10E-04	Regulation of glucose transmembrane transport	KO_down_OE_up	Prkcb, Adipoq, Aoc3, Ffar4

## Discussion

Using two novel skeletal muscle-specific mouse models, one of PAK1 depletion and the other of PAK1 enrichment, this study fills key gaps in our understanding about the requirement for skeletal muscle PAK1 in regulating glucose homeostasis. Our data show that skeletal muscle PAK1 levels positively correlate with peripheral insulin sensitivity and glucose tolerance. These new mouse models are inducible, such that loss or gain of PAK1 was limited to adult mice, obviating confounds associated with potential developmental effects. The tissue specificity of our new mouse models allows us to focus on the role of PAK1 in skeletal muscle without disrupting its action in other tissues. SkmPAK1-iKO mice exhibit peripheral insulin resistance, defects in hindlimb skeletal muscle insulin-stimulated GLUT4 translocation, and show whole body glucose intolerance. F-actin remodeling is a prerequisite for GLUT4 vesicle translocation and glucose uptake in L6 myoblasts (44) and in mature skeletal muscle (45); the identification of defects in insulin-stimulated GLUT4 translocation and insulin intolerance in the skmPAK1-iKO mice provide *in vivo* support for this concept. Indeed, IPA3, a known pharmacological agent which specifically inhibits Rac1-induced PAK1 activation, completely abolished insulin-induced F-actin remodeling, GLUT4 translocation, and glucose uptake into L6 skeletal muscle cells (35). Similarly, L6 myotubes exposed to chronic hyperinsulinemia displayed a phenotype in which both GLUT4 accumulation at the PM and glucose uptake were significantly reduced concomitant with loss of cortical actin (17). Indeed, future studies using the new skmPAK1-iKO described here will aid in the understanding of the complex signaling cascades involved in the dynamic regulation of GLUT4 vesicle translocation to the PM. In addition, muscle-specific Rac1 KO mice exhibit significantly reduced glucose tolerance, consistent with this PAK1-centric model, while acknowledging the potential for other Rac1-based mechanisms regulating glucose uptake (13). Unexpectedly, our skmPAK1-iKO mice also show a delayed increase in plasma insulin content after glucose challenge (injection). Using our unique skmPAK1-iOE mice, coupled with findings from studies using CM, we have unveiled a putative tissue crosstalk pathway between skeletal muscle and pancreatic β-cells, which is regulated by skeletal muscle PAK1. RNA-Seq studies using whole hindlimb skeletal muscle from iKO and iOE mice identified differentially expressed gene signatures that suggest a model whereby PAK1 enrichment in muscle triggers release of factor(s) associated with positive effects upon glucose homeostasis.

Muscle-derived signals mediate the crosstalk of muscle with target tissues in an autocrine, paracrine, and endocrine manner. More recently, attention has been raised to a role for skeletal muscle in regulating β-cell mass and function. Conditioned media studies using human T2D skeletal muscle cells have demonstrated that myokines from the skeletal muscle can modulate GSIS in INS-1 832/13 β-cells (46); the presence of myokines could be an explanation for why GSIS was boosted in INS-1 832/13 β-cells treated with CM collected from myotubes overexpressing PAK1. In addition, exercise training has been shown to increase GSIS (47). The loss or gain of PAK1 in skeletal muscle is also associated with inhibition or enhancement of muscle contraction during exercise (5, 48) respectively. For example, IL-6 is a well-characterized myokine that is increased by exercise in skeletal muscle; when released, IL-6 elicits crosstalk with β-cells and potentiates GSIS (49). In addition, myotubes can release the enzyme adenylate kinase 1 which synthesizes extracellular ATP (50), as well as redox proteins that may have systemic redox signaling and antioxidant functions that in/directly benefit the pancreatic islet β-cells (51). Even though there are several lines of evidence suggesting that skeletal muscle is an active endocrine organ (52, 53) with the ability to modify the function of other cells and tissues including islet β-cells, the identity of exact mediator(s) responsible for increased GSIS are currently unknown. Although, many factors, including myokines (46) metabolites (54), microRNAs (55), and factors packaged in exosomes (56) are secreted by skeletal muscle, it is possible that a single factor or a combination of multiple factors contributes to the enhanced GSIS observed.

PAK1 is known to be capable of transiting into the nucleus to transactivate genes (42), and hence it was not surprising to detect a spectrum of gene changes that showed coordinated changes in KO versus OE in the RNAseq experiments using primary skeletal muscle tissue. Our RNAseq data demonstrated that the genes involved in negatively regulating apoptosis were significantly reduced in skmPAK1-iKO mice, in contrast to skmPAK1-iOE, where the negative regulators of apoptosis were significantly elevated. In this context it is germane to point out that Adipoq (Adiponectin) and Ptgs2 (Prostaglandin-Endoperoxide Synthase 2, aka cyclooxygenase-2, COX-2) may be under the regulation of PAK1. Adiponectin has been reported to be expressed by rodent skeletal muscle fibers/cells and exert positive effects on glucose uptake (57, 58). Furthermore, adiponectin’s antiapoptotic action in cardiac cells is mediated *via* Cox2 (59). The direct relationship of PAK1 in modulating adiponectin and Cox2 is still unclear, and investigations are underway to dissect these pathways in our laboratory. In addition to identifying the two known skeletal muscle related genes as top hits, APOLD1 (60) and SCD1 (61), SCD1 is also as well described adipose factor (62), and several other adipocyte-related genes were also identified (e.g., PLIN1 and CIDEC) (63, 64). That these adipocyte-related genes changed, yet only skeletal muscle PAK1 levels were targeted, might suggest local paracrine changes within the skeletal muscle tissue, tissue which contains numerous cell types, including adipocytes, beyond skeletal muscle cells. Skeletal muscle intramyocellular lipids (IMCLs) serve as a rapid fuel source for mitochondrial fat oxidation, during exercise (65). ARPC1B expression changes showed correlative trends, increasing in skmPAK1-iOE vs control, and decreasing in skmPAK1-iKO vs the matched control, although did not reach statistical significances. Furthermore, our GO analysis identified regulation of actin cytoskeleton organization and actin filament-based processes in skmPAK1-iOE but did not reach statistical significance. Nonetheless, our conditioned media experiments point to factor(s) released from PAK1-overexpressing myotubes that when applied to β-cells, enhanced the function of those β-cells. Further proteomic, metabolomic and transcriptomic studies will be required to identify these factor(s). In addition, it will be important in future studies to factor in the consideration of muscle fiber type, since there are studies suggesting that myotubes derived from different fiber types present distinct gene signatures (66, 67).

We and others originally demonstrated that PAK1 is required to maintain glucose homeostasis from studies of the classic whole body PAK1 KO mice (10, 12). To our surprise, another report using the original whole body PAK1 KO contradicts the findings of the earlier studies of that mouse model (11). However, all reports using the older constitutive whole body PAK1 KO model are complicated by the potential and possibly differential impacts upon development, as well as the deletion of PAK1 occurring in all tissues and the potential for differential compensatory mechanisms involved. Our current studies using the new skeletal muscle specific deletion, and deleted inducibly only in adult mice, circumvents these prior caveats. Potential redundancy of PAK1/2 proteins may also be involved, since group I PAK’s are highly conserved and targeting a specific isoform may prompt compensatory benefit from other isoform. For example, PAK2 was implicated in insulin-stimulated glucose uptake into isolated glycolytic extensor digitorum longus muscle, alone or crossed to the original whole body PAK1 KO mice (11). However, the mice were on a mixed genetic background of FVB and C57BL6J, which has the capacity to differentially impact metabolism and glucose homeostatic output data. Moreover, those PAK1: PAK2 double KO mice exhibited myopathy with significant alterations in mitochondrial morphology, complicating interpretations. PAK2 knockdown in L6 skeletal muscle cells was without effect upon insulin-stimulated GLUT4 translocation in L6 muscle cells (35). Until the PAK2 KO is enriched on the C57BL6J background, and generated with an inducible skeletal muscle specific Cre, a requirement for muscle PAK2 in glucose homeostasis in adult mice remains unclear. Moreover, whether PAK2 is required for insulin-stimulated GLUT4 translocation, as we show in the current study that PAK1 is required for, remains undetermined.

While our studies of human PAK1 protein levels here show decreased PAK1 in T2D vs nondiabetic control muscle tissue, using muscles from quadriceps or ‘leg’ (information from NDRI, possibly of mixed muscle fiber types (14)), another group examined PAK1 protein levels in vastus lateralis (VL) muscle in human biopsy samples (13), where they observed a 27% increase in PAK1 levels in T2D compared with that of lean or obese controls. Given that VL was suggested to harbor greater levels of PAK1 than soleus in humans (13), and that our samples were from ‘leg’, which may have included soleus, this may be a case of skeletal muscle heterogeneity. Skeletal muscle heterogeneity is attributed to several characteristics, such as inherent differences in muscle fiber type, and in particular, differences in myocellular contractile function between the VL and soleus (68). It remains a possibility that differing fiber types, respond differentially to diabetogenic stimuli that have been shown to impinge upon PAK1 protein levels. Given the contradictory results reported so far in human skeletal muscles of people with T2D, further studies would be necessary in human skeletal muscle.

Overall, we have identified novel roles of skeletal muscle PAK1 as a regulator of glucose homeostasis and a mediator of tissue crosstalk with pancreatic β-cells. Additionally, PAK1 levels appear to lead to significant changes in gene regulation. We propose that targeting skeletal muscle PAK1 may be of therapeutic benefit for T2D and that PAK1 may regulate an important new connection between glucose tolerance and lipid storage, delivery and metabolism.

## Data Availability Statement

The datasets presented in this study can be found in online repositories. The names of the repository/repositories and accession number(s) can be found below: NCBI GEO, accession no: GSE191000.

## Ethics Statement

The animal study was reviewed and approved by The Institutional Animal Care and Use Committee of City of Hope National Medical Center (Duarte, CA, USA; approval #15023).

## Author Contributions

KEM, MA, and RV collected data and analysis, contributed to discussion, and wrote/edited manuscript. RT and VAS performed *in vivo* mouse studies, contributed to discussion, and edited the manuscript. MA and EO generated the skmPAK1-iOE and skmPAK1-iKO mouse models, contributed to discussion, and edited the manuscript. RV and JH performed conditioned media experiments, *ex vivo* GSIS and insulin content assays, contributed to discussion and edited the manuscript. SB performed the bioinformatics analysis. EMM, PAG, and CZ performed *in vivo* studies and edited the manuscript. SMY assisted with generating the skmPAK1-iKO model, contributed to discussion, and edited the manuscript. JSE contributed to discussion and edited the manuscript. DCT conceived of the study, contributed to discussion, and wrote/reviewed/edited the manuscript. DCT is the guarantor of this work and, as such, had full access to all the data in the study and takes responsibility for the integrity of the data and the accuracy of the data analysis. All authors read and approved the final version of the manuscript.

## Funding

This study was supported by grants from the National Institutes of Health (DK067912, DK112917 and DK102233 to DCT; DK114222 and DK097512 to JSE) and postdoctoral fellowships from the Larry L. Hillblom Foundation (#2019-D-015-FEL to RT, #2020-D-018-FEL to JH).

## Conflict of Interest

SMY is employed by the company Eli Lilly & Company.

The remaining authors declare that the research was conducted in the absence of any commercial or financial relationships that could be construed as a potential conflict of interest.

## Publisher’s Note

All claims expressed in this article are solely those of the authors and do not necessarily represent those of their affiliated organizations, or those of the publisher, the editors and the reviewers. Any product that may be evaluated in this article, or claim that may be made by its manufacturer, is not guaranteed or endorsed by the publisher.
