# Supplementary material for: Changes in Skeletal Muscle PAK1 Levels Regulate Tissue Crosstalk to Impact Whole Body Glucose Homeostasis
Source: Front Endocrinol (Lausanne). 2022 Feb 11;13:821849. doi: 10.3389/fendo.2022.821849 (PMC8881144; doi:10.3389/fendo.2022.821849)
Supplement: Supplementary file 1 [file DataSheet_1.docx]

**Supplemental Information**

**CHANGES IN SKELETAL MUSCLE PAK1 LEVELS REGULATE TISSUE CROSSTALK TO IMPACT WHOLE BODY GLUCOSE HOMEOSTASIS**

Karla E. Merz^1^*, Ragadeepthi Tunduguru^2^*, Miwon Ahn^1^*, Vishal A. Salunkhe**^3^***, Rajakrishnan Veluthakal^1^, Jinhee Hwang^1^, Supriyo Bhattacharya^4^, Erika M. McCown^1^, Pablo A. Garcia^1^, Chunxue Zhou^1^, Eunjin Oh^1^, Stephanie M. Yoder^5^, Jeffrey S. Elmendorf^6^, and Debbie C. Thurmond^1^

*Contributed equally to this work

^1^Department of Molecular & Cellular Endocrinology, Arthur Riggs Diabetes and Metabolism Research Institute of City of Hope, Duarte, CA.

^2^Department of Diabetes Complications and Metabolism, Arthur Riggs Diabetes and Metabolism Research Institute of City of Hope, Duarte, CA.

^3^Sahlgrenska Academy, Institute of Neuroscience and Physiology, Metabolism research unit, University of Gothenburg, Gothenburg, Sweden.

^4^Division of Translational Bioinformatics, City of Hope, Duarte, CA

^5^Eli Lilly & Company, Indianapolis, IN

^6^Department of Anatomy, Cell Biology and Physiology, Center for Diabetes and Metabolic Disease, Indiana University School of Medicine, Indianapolis, IN

**Corresponding Author:** Debbie C. Thurmond, Ph.D.

Department of Molecular & Cellular Endocrinology,

City of Hope Beckman Research Institute, Duarte CA 91010

E-mail: [dthurmond@coh.org](mailto:dthurmond@coh.org)

Phone: 626.218.0190

**Table of Contents:**

**Supplemental Figure 1 ……………………………………………………………………S2**

**Supplemental Figure 2 ……………………………………………………………………S3**

**Supplemental Figure 3 ……………………………………………………………………S4**

**Supplemental Figure 4 ……………………………………………………………………S5**

**Supplemental Table 1 ……………………………………………………………………S6**

**Supplemental Table 2 ……………………………………………………………………S7**

**Supplemental Table 3 ……………………………………………………………………S8**

**Supplemental Table 4 ……………………………………………………………………S9**

**Supplemental Table 5 ……………………………………………………………………S10-11**

**Supplemental References……………………………………………………………....…S12Supplemental Figure 1.**

**
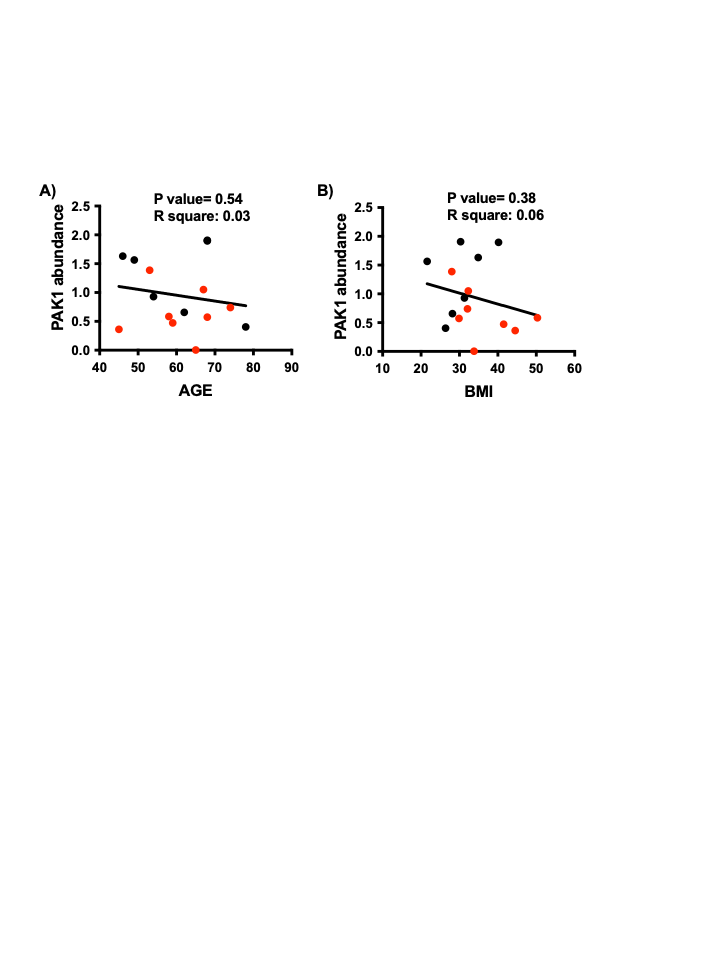
**

**Supplemental Figure 1. Evaluations of PAK1 abundance correlations in human skeletal muscle.** Correlation of human skeletal muscle PAK1 protein abundance correlation with (A) Age, and (B) BMI, n=15 (ND;n=7, T2D;n=8), linear regression analysis.

**Supplemental Figure 2.**

**
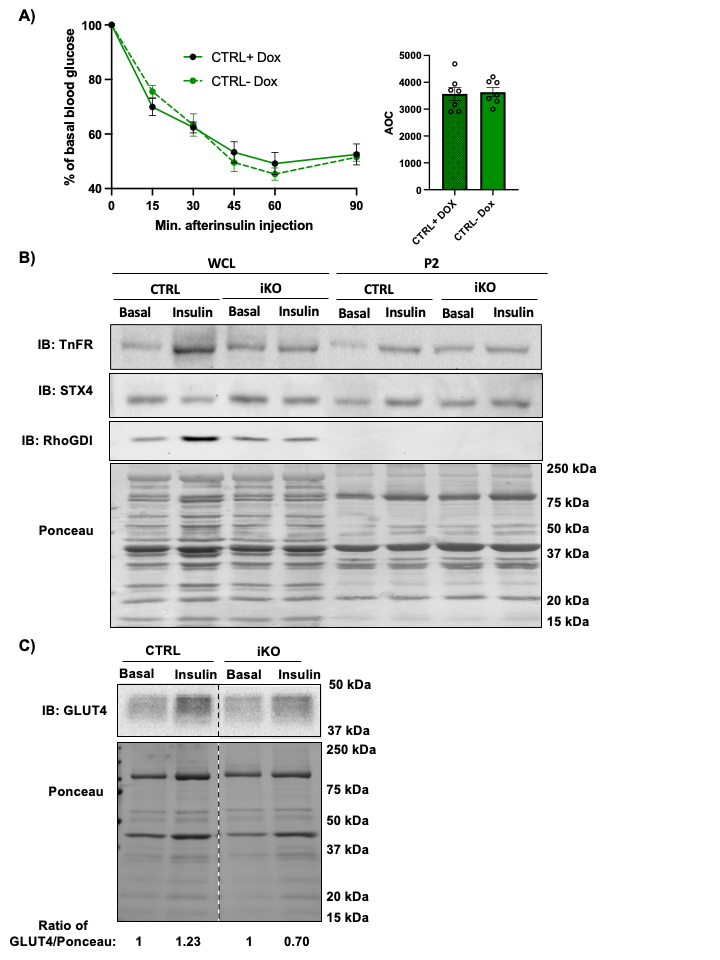
**

**Supplemental Figure 2. DOX control data and GLUT4 fractionation control data.** (A) IPGTT and AUC are similar in CTRL mice without and with DOX treatment in male mice (n=7 per group; p>0.05. (B) Purity of the GLUT4 P2 fraction. P2 fractions were assessed for purity using the fraction markers TnFR and STX4, as described previously (1, 2). RhoGDI, an established cytosolic protein (3, 4) was undetectable in the P2 fraction despite its presence in the starting input skm cell lysate (WCL) from which the P2 fraction was derived. (C) Representative set of GLUT4 immunoblots from P2 fractions used in quantitative analyses. GLUT4 band density was normalized for differences in gel loading using Ponceau S stained protein content.

**Supplemental Figure 3.**

**
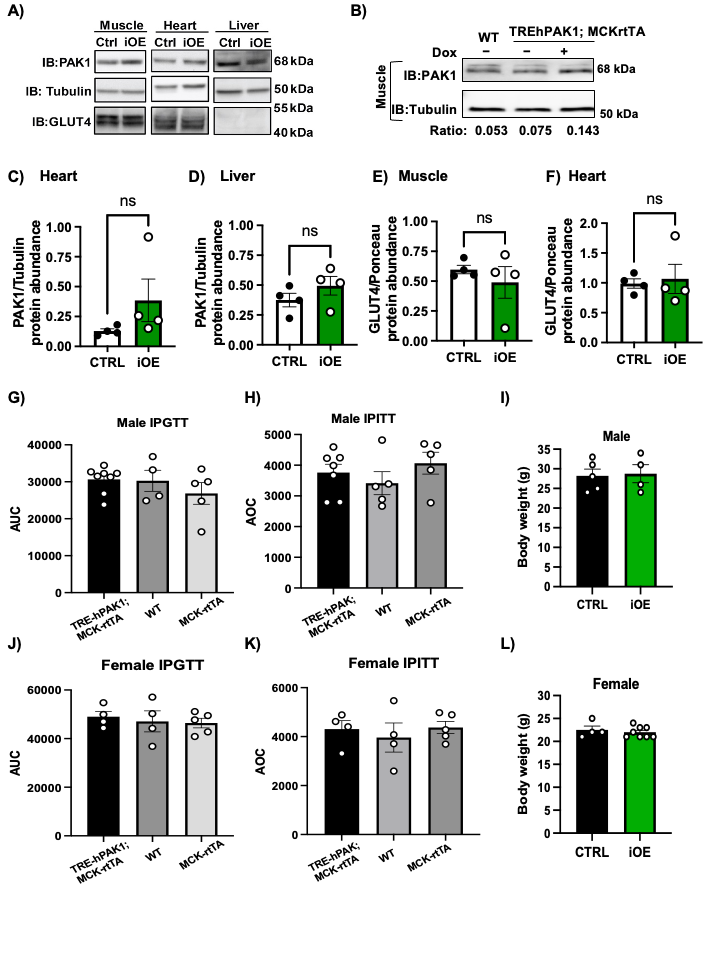
**

**Supplemental Figure 3. Evaluation of skm PAK1 iOE mouse tissue protein abundances, glucose tolerance and insulin tolerance for appropriate Doxycycline (Dox)-induction of PAK1 and lack of off-target effects of Dox. (A)** PAK1 abundance in mouse skeletal muscle, heart, and liver in Ctrl (non-Dox-induced TRE-PAK1; Mck-rtTA) and skeletal muscle-specific PAK1-enriched (iOE=Dox-induced TRE-PAK1;Mck-rtTA) male mice. Data are representative of n=4 per group. (B) PAK1 abundance in non-Dox-treated wild-type (WT) and TRE-hPAK1:Mck-rtTA mice as well as mice with skeletal muscle-specific PAK1 enrichment (Dox-treated TRE-hPAK1:Mck-rtTA). PAK1 protein abundance quantification in heart (C) and liver (D). GLUT4 protein abundance in muscle (E) and heart (F). Student’s T-test analyses were used in panels C-F; no significant differences (ns) were detected. Area under the curve (AUC) values for the intraperitoneal glucose tolerance test (IPGTT) in non-Dox-induced TRE-hPAK1;Mck-rtTA, Dox-induced WT, and Dox-induced Mck-rtTA mice; (G) male and (J) female, n=4-7 per group. Area over the curve (AOC, baseline set to 100%) for the intraperitoneal insulin tolerance test (IPITT) in non-Dox-induced TRE-hPAK1:Mck-rtTA, Dox-induced WT, and Dox-induced Mck-rtTA mice; (H) male and (K) female, n=4-7 per group. Body weight of male (I) and female (L) CTRL and skmPAK1-iOE mice, n=4-7 per group.

**Supplemental Figure 4.
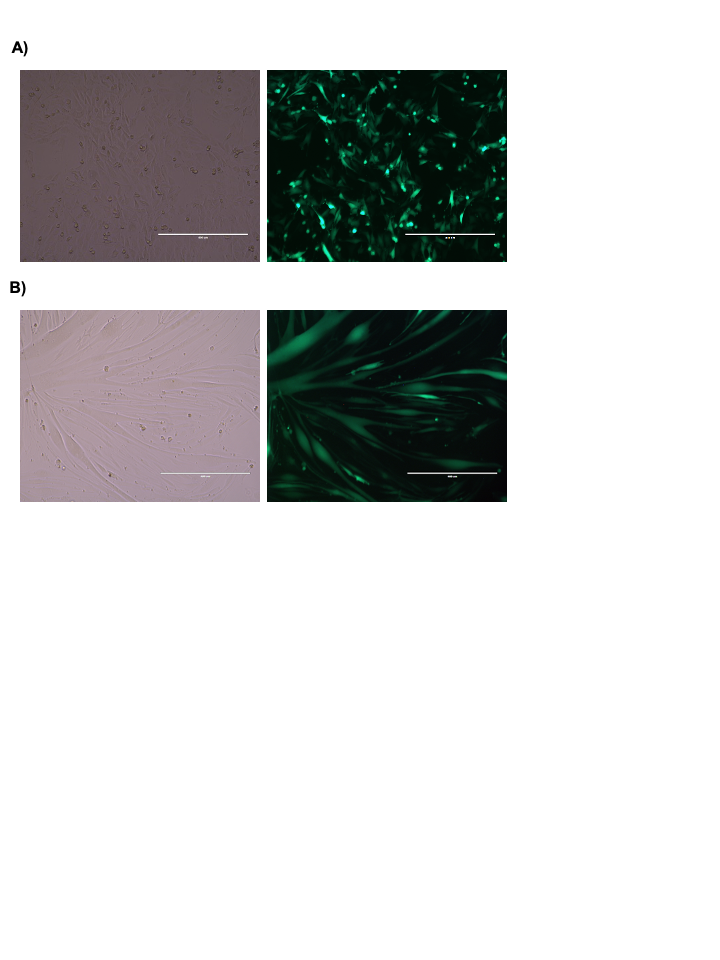
**

**Supplemental Figure 4.** Representative images of transfection efficiency of myoblasts (A), and transduction efficiency in myotubes (B).

|  |  |  |  |  |  |  |
| --- | --- | --- | --- | --- | --- | --- |
| **Sample** | **NDRI#** | **Age** | **Race** | **Gender** | **BMI** | **Condition** |
| 1 | ND09704 | 68 | Caucasian | M | 30.3 | ND |
| 2 | ND09743 | 46 | Caucasian | M | 34.9 | ND |
| 3 | ND09744 | 62 | Caucasian | M | 28.2 | ND |
| 4 | ND09749 | 49 | Caucasian | F | 21.6 | ND |
| 5 | ND09703 | 68 | Caucasian | F | 29.9 | T2D |
| 6 | ND09705 | 65 | Caucasian | M | 33.8 | T2D |
| 7 | ND09706 | 45 | Caucasian | M | 44.5 | T2D |
| 8 | ND09754 | 67 | Caucasian | M | 32.3 | T2D |
| 9 | ND10105 | 53 | Caucasian | F | 28 | T2D |
| 10 | ND13347 | 78 | Caucasian | M | 26.4 | ND |
| 11 | ND13230 | 68 | Caucasian | F | 40.2 | ND |
| 12 | ND13216 | 54 | Caucasian | M | 31.3 | ND |
| 13 | ND13214 | 74 | Caucasian | M | 32.1 | T2D |
| 14 | ND13363 | 59 | Caucasian | M | 41.5 | T2D |
| 15 | ND13239 | 58 | Caucasian | M | 50.3 | T2D |
|  |  |  |  |  |  |  |

**Supplemental Table 1**. Non- diabetic and T2D human skeletal muscle donor information.

ND= Non-diabetic individuals; T2D= Type 2 diabetic individuals; BMI= Body mass index;

M= Male, F= Female; NDRI# = National disease research interchange (NDRI) sample number, provided muscle source as quadriceps or ‘leg muscle’.

**Supplemental Table 2.** Tissue and body weights of chow-fed CTRL and skmPAK1-iKO male mice.

|  | **CTRL (n=4)** | **fl/fl;Cre- :Dox (n=6)** | **iKO (n=9)** |
| --- | --- | --- | --- |
| **Body weight (g)** | 31.4 $\pm$ 0.31 | 30.$3 \pm$ 0.97 | 31.$6 \pm$0.71 |
| **Tissue weights (% of BW)** | | | |
| **Brain** | 1.55 $\pm$0.06 | 1.69 $\pm$ 0.04 | 1.48 $\pm$0.06 |
| **Skeletal Muscle** | 1.04 $\pm$0.11 | 1.11 $\pm0.06$ | 1.12 $\pm$0.05 |
| **Fat** | 2.20 $\pm$0.22 | 1.51 $\pm0.21$ | 1.61 $\pm$0.31 |
| **Spleen** | 0.28 $\pm$0.03 | 0.31 $\pm0.05$ | 0.31 $\pm$0.04 |
| **Lungs** | 0.59 $\pm$0.06 | 0.82 $\pm0.10$ | 0.68 $\pm$0.06 |
| **Liver** | 4.81 $\pm$0.33 | 4.48 $\pm0.26$ | 4.46 $\pm$0.10 |
| **Kidney** | 1.49 $\pm$0.06 | 1.50 $\pm$ 0.12 | 1.60 $\pm$0.06 |
| **Heart** | 0.65 $\pm$0.05 | 0.84 $\pm0.09$ | 0.73 $\pm$0.04 |
| **Pancreas** | 0.60 $\pm$0.04 | 1.01 $\pm0.09$ | 0.77 $\pm$0.07 |

Data represent the average ± SEM of 4-6 month-old male mice. CTRL mice (fl/fl; cre+, not induced), PAK1 fl/fl; Cre-, with Dox, and iKO (PAK1 fl/fl; cre+, Dox-induced) skeletal muscle weights represent the muscle from the left hindlimb only. No statistically significant differences were observed. BW, body weight.

|  | **CTRL (n=5)** | **sTg + Dox (n=5)** | **iOE (n=4)** |
| --- | --- | --- | --- |
| **Body weight (g)** | 28.2 $\pm$ 1.72 | 29.4 $\pm$ 1.57 | 28.8 $\pm$2.29 |
| **Tissue weights (% of BW)** | | | |
| **Skeletal Muscle** | 1.78 $\pm$0.20 | 1.82 $\pm0.27$ | 2.18 $\pm$0.22 |
| **Fat** | 1.04 $\pm$0.19 | 0.75 $\pm0.19$ | 0.73 $\pm$0.11 |
| **Spleen** | 0.35 $\pm$0.08 | 0.28 $\pm0.05$ | 0.28 $\pm$0.03 |
| **Lungs** | 0.67 $\pm$0.06 | 0.55 $\pm0.02$ | 0.71 $\pm$0.04 |
| **Liver** | 4.50 $\pm$0.10 | 4.18 $\pm0.33$ | 4.01 $\pm$0.14 |
| **Kidney** | 1.49 $\pm$0.03 | 1.49 $\pm$ 0.08 | 1.42 $\pm$0.06 |
| **Heart** | 0.73 $\pm$0.06 | 0.53 $\pm0.04$ | 0.65 $\pm$0.08 |
| **Pancreas** | 0.83 $\pm$0.16 | 0.58 $\pm0.09$ | 0.68 $\pm$0.10 |

**Supplemental Table 3.** Tissue and body weights of chow-fed CTRL and skmPAK1-iOE male mice.

Data represent the average ± SEM of 4-6 month-old male mice. CTRL (TRE-PAK1; Mck-rtTA), sTg (TRE-PAK1 or Mck-rtTA with Dox), and iOE (TRE-PAK1; Mck-rtTA Dox-induced) skeletal muscle isolated from both hindlimbs. No statistically significant differences observed.

|  | **CTRL (n=4)** | **sTg + Dox (n=14)** | **iOE (n=8)** |
| --- | --- | --- | --- |
| **Body weight (g)** | 22.5 $\pm$ 0.87 | 23.9 $\pm$ 0.53 | 22.0 $\pm$0.42 |
| **Tissue weights (% of BW)** | | | |
| **Skeletal Muscle** | 1.75 $\pm$0.39 | 1.55 $\pm0.12$ | 1.66 $\pm$0.14 |
| **Fat** | 1.78 $\pm$0.41 | 1.16 $\pm0.12$ | 0.85 $\pm$0.09 |
| **Spleen** | 0.27 $\pm$0.04 | 0.32 $\pm0.03$ | 0.30 $\pm$0.05 |
| **Lungs** | 0.72 $\pm$0.06 | 0.65 $\pm0.03$ | 0.68 $\pm$0.05 |
| **Liver** | 4.48 $\pm$0.31 | 3.74 $\pm0.13$ | 4.13 $\pm$0.22 |
| **Kidney** | 1.34 $\pm$0.09 | 1.43 $\pm$ 0.05 | 1.45 $\pm$0.02 |
| **Heart** | 0.72 $\pm$0.03 | 0.46 $\pm0.02$ | 0.76 $\pm$0.21 |
| **Pancreas** | 0.94 $\pm$0.16 | 0.72 $\pm0.05$ | 0.75 $\pm$0.08 |
| **Brain** | 1.15 $\pm$0.38 | 1.01 $\pm0.14$ | 1.06 $\pm$0.26 |

**Supplemental Table 4.** Tissue and body weights of chow-fed CTRL and skmPAK1-iOE female mice.

Data represent the average ± SEM of 4-6 month-old female mice. CTRL (TRE-PAK1; Mck-rtTA) and PAK1 (TRE-PAK1; Mck-rtTA Dox-induced) skeletal muscle isolated from both hindlimbs. No statistically significant differences observed.

**Supplemental Table 5.** Gene expression levels found to be the most strongly associated with PAK1 expression levels showing overlap between the skmPAK1-iKO and skmPAK1-iOE group.

|  |  | iOE | | iKO | |
| --- | --- | --- | --- | --- | --- |
| Gene ID | Description | Fold Change | P-value | Fold Change | P-value |
| PLIN1 | Perilipin-1: A modulator of adipocyte lipid metabolism. | 1.78 | 4.37E-02 | -2.85 | 2.93E-13 |
| NNAT | Neuronatin: Regulation of ion channels during brain development. | 2.89 | 2.33E-02 | -3.48 | 1.97E-09 |
| PTK2B | Protein tyrosine kinase 2-β: Calcium-induced regulation of ion channels and activation of the MAP kinase signaling pathway, actin reorganization and cell polarization. | 3.92 | 6.97E-04 | -2.31 | 5.63E-15 |
| ARXES2 | Adipocyte related x-chromosome expressed sequence 2: Fat cell differentiation, ER protein targeting and peptide processing. Required for adipogenesis. | 3.73 | 2.10E-04 | -2.57 | 2.04E-06 |
| VPREB3 | V-Set Pre-B Cell Surrogate Light Chain 3: Play role in B-cell maturation, assembly of the pre-B cell receptor (pre-BCR) and immunoglobulin production. | 3.92 | 1.85E-02 | -2.55 | 2.99E-04 |
| PCK1 | Cytosolic Phosphoenolpyruvate carboxykinase: Catalyzes the reversible decarboxylation and phosphorylation of oxaloacetate (OAA) and acts as the rate-limiting enzyme in gluconeogenesis. | 2.71 | 1.12E-02 | -2.31 | 5.15E-07 |
| SCD1 | Stearoyl-CoA desaturase1: A key enzyme in fatty acid metabolism that produces the monounsaturated fatty acid oleic acid from the saturated fatty acid stearic acid. | 3.03 | 4.20E-04 | -2.19 | 2.79E-03 |
| SLC1A3 | Solute Carrier Family 1 Member 3: Sodium-dependent, high-affinity amino acid transporter that mediates the uptake of L-glutamate, L-aspartate and D-aspartate. | 2.53 | 3.74E-04 | -1.92 | 1.30E-06 |
| KIF21B | Kinesin Family Member 21B: ATP-dependent microtubule-based motor protein that is involved in the synapse function, learning and memory. | 2.77 | 3.36E-03 | -1.88 | 1.12E-20 |
| RFC5 | Replication factor C subunit 5: Functions in cell replication and proliferation/chromatin remodeling. | 2.53 | 2.10E-02 | -1.52 | 2.06E-02 |
| KBTBD11 | Kelch repeat and BTB domain-containing protein 11: A negative regulator of osteoclastogenesis, possibly involved in E3 ubiquitin ligase-mediated events. | 2.64 | 5.51E-03 | -1.55 | 5.94E-08 |
| NSG2 | Neuronal vesicle trafficking associated 2: Important for clathrin light chain binding and coat assembly (endosomal transport). | 3.56 | 2.31E-03 | -1.62 | 1.83E-10 |
| CIDEC | Cell Death Inducing DFFA Like Effector C: Promotes lipid droplet formation in adipocytes; may mediate adipocyte apoptosis. | 3.16 | 4.36E-05 | -1.66 | 2.03E-02 |
| NRGN | Neurogranin: A substrate of protein kinase C pathways involved in synapse development and remodeling. Binds calmodulin when calcium is absent. | 3.12 | 1.53E-02 | -1.58 | 3.53E-02 |
| SLC17A9 | Solute carrier family 17 member 9: Participates in the vesicular uptake, storage, and secretion of adenosine triphosphate (ATP) and other nucleotides in adrenal chromatin granules and synaptic vesicles. | 2.81 | 5.42E-03 | -1.46 | 8.17E-04 |
| DUSP6 | Dual specificity protein phosphatase 6: Inactivate target kinase by dephosphorylating both the phosphoserine/threonine and phosphotyrosine residues. | 2.93 | 2.04E-05 | -1.49 | 3.15E-03 |
| SLC38A1 | Solute Carrier Family 38 Member 1: Sodium dependent amino acid transporter: Mediates glutamine transport. | 3.1 | 2.09E-03 | -1.21 | 8.17E-04 |
| EGR3 | Early growth response protein 3: Involved in transcriptional regulation of genes in controlling biological rhythm. | 3.1 | 8.18E-04 | -1.4 | 3.71E-04 |
| APOLD1 | Apolipoprotein L domain-containing protein 1: Involved in lipid binding, angiogenesis, cell differentiation and lipid transport. | 3.1 | 8.73E-05 | -1.36 | 3.11E-02 |
| APLP1 | Amyloid-like Beta Precursor Like protein 1: Regulates neurite growth by binding heparin and collagen. Binds zinc and copper. Enhances neuronal apoptosis. | 3.18 | 4.27E-03 | -1.36 | 4.95E-02 |
| TUBB3 | Tubulin beta-3 chain: A major constituent of microtubules. Involved in axon guidance and maintenance. | 3.18 | 4.95E-02 | -1.34 | 3.75E-02 |

**Supplemental References**

1. Zhou, M., Sevilla, L., Vallega, G., Chen, P., Palacin, M., Zorzano, A., Pilch, P. F., and Kandror, K. V. (1998) Insulin-dependent protein trafficking in skeletal muscle cells. *Am J Physiol* **275**, E187-E196

2. Oh, E., Spurlin, B. A., Pessin, J. E., and Thurmond, D. C. (2005) Munc18c heterozygous knockout mice display increased susceptibility for severe glucose intolerance. *Diabetes* **54**, 638-647.

3. Garcia-Mata, R., Boulter, E., and Burridge, K. (2011) The 'invisible hand': regulation of RHO GTPases by RHOGDIs. *Nat Rev Mol Cell Biol* **12**, 493-504

4. Moller, L. L. V., Klip, A., and Sylow, L. (2019) Rho GTPases-Emerging Regulators of Glucose Homeostasis and Metabolic Health. *Cells* **8**, 434.
