# Supplementary material for: Changes in Skeletal Muscle PAK1 Levels Regulate Tissue Crosstalk to Impact Whole Body Glucose Homeostasis
Source: Front Endocrinol (Lausanne). 2022 Feb 11;13:821849. doi: 10.3389/fendo.2022.821849 (PMC8881144; doi:10.3389/fendo.2022.821849)

Figure 1 A):

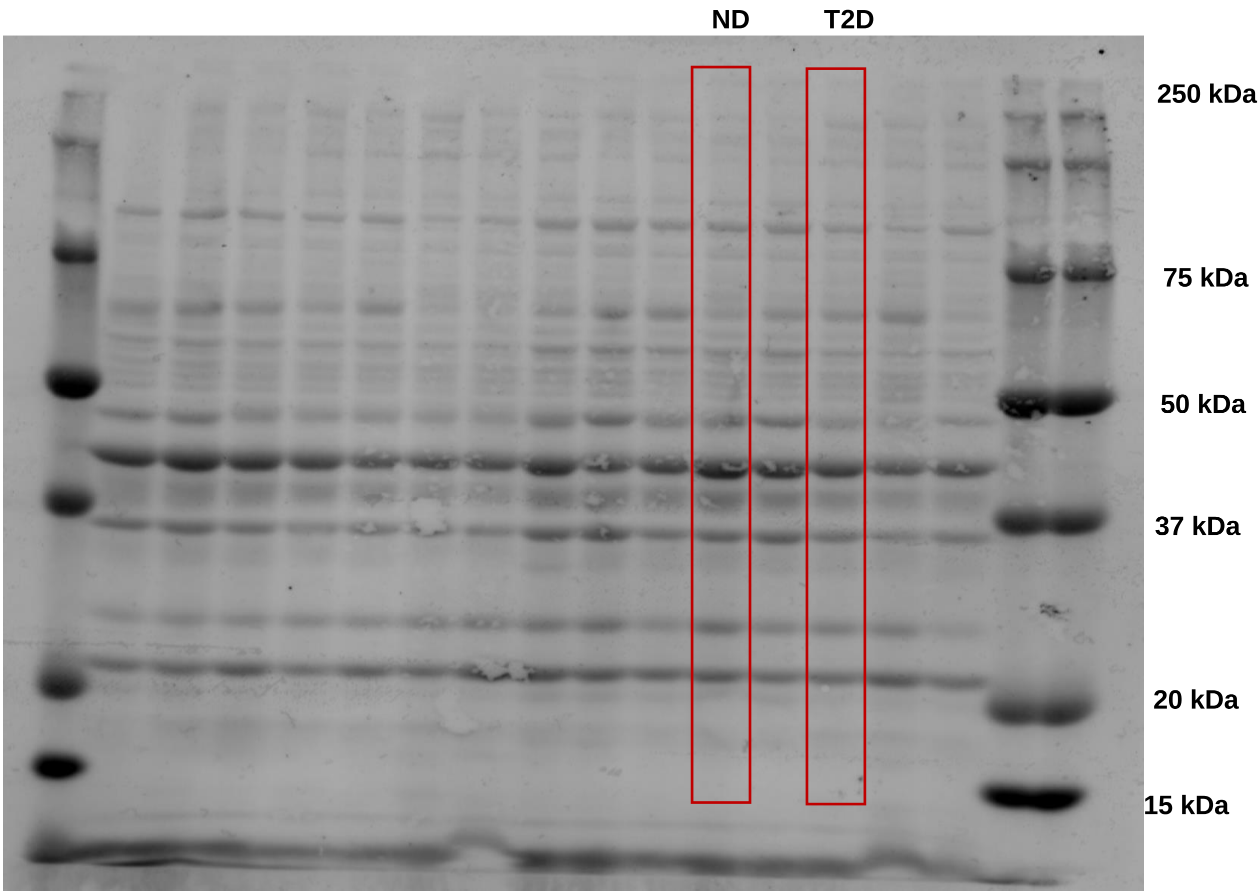

Figure 2 B):

Skeletal muscle

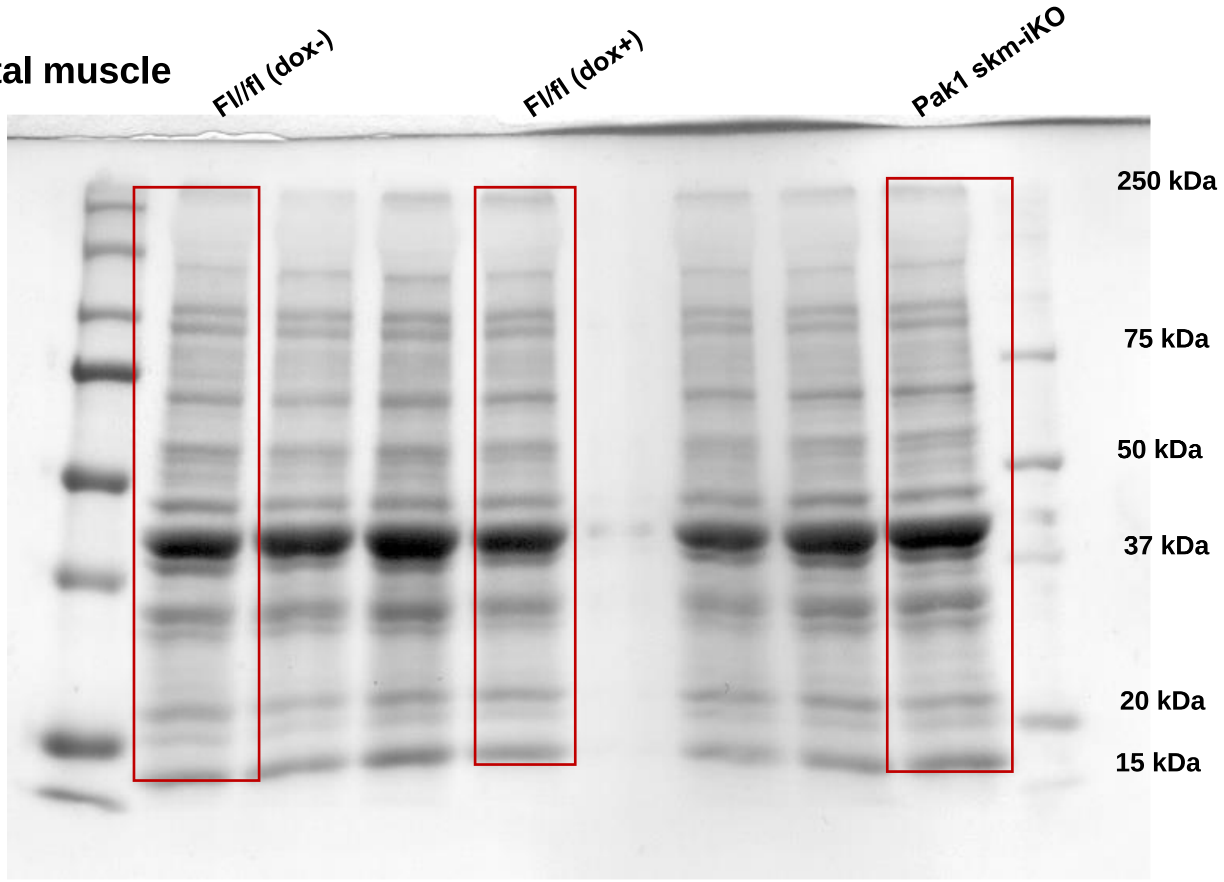

Heart

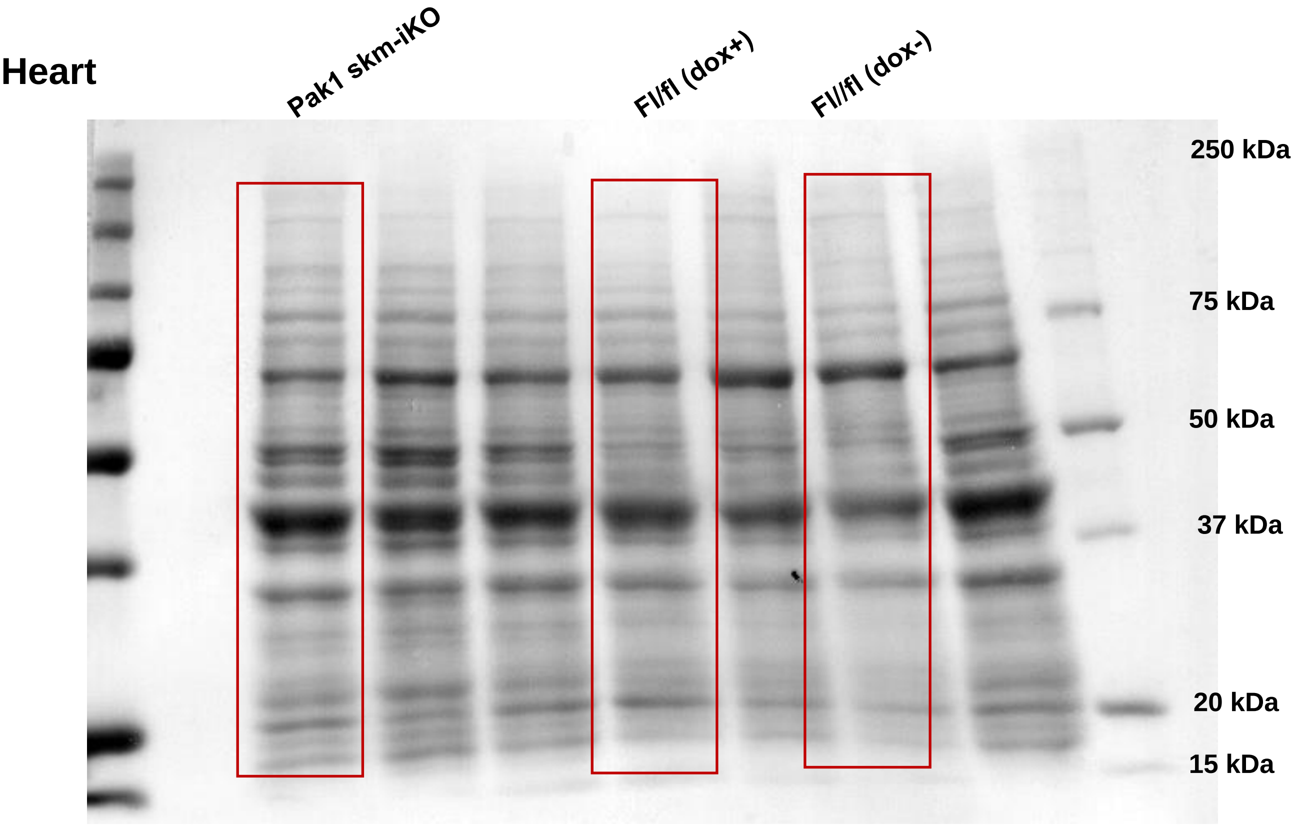

Figure 3 B):

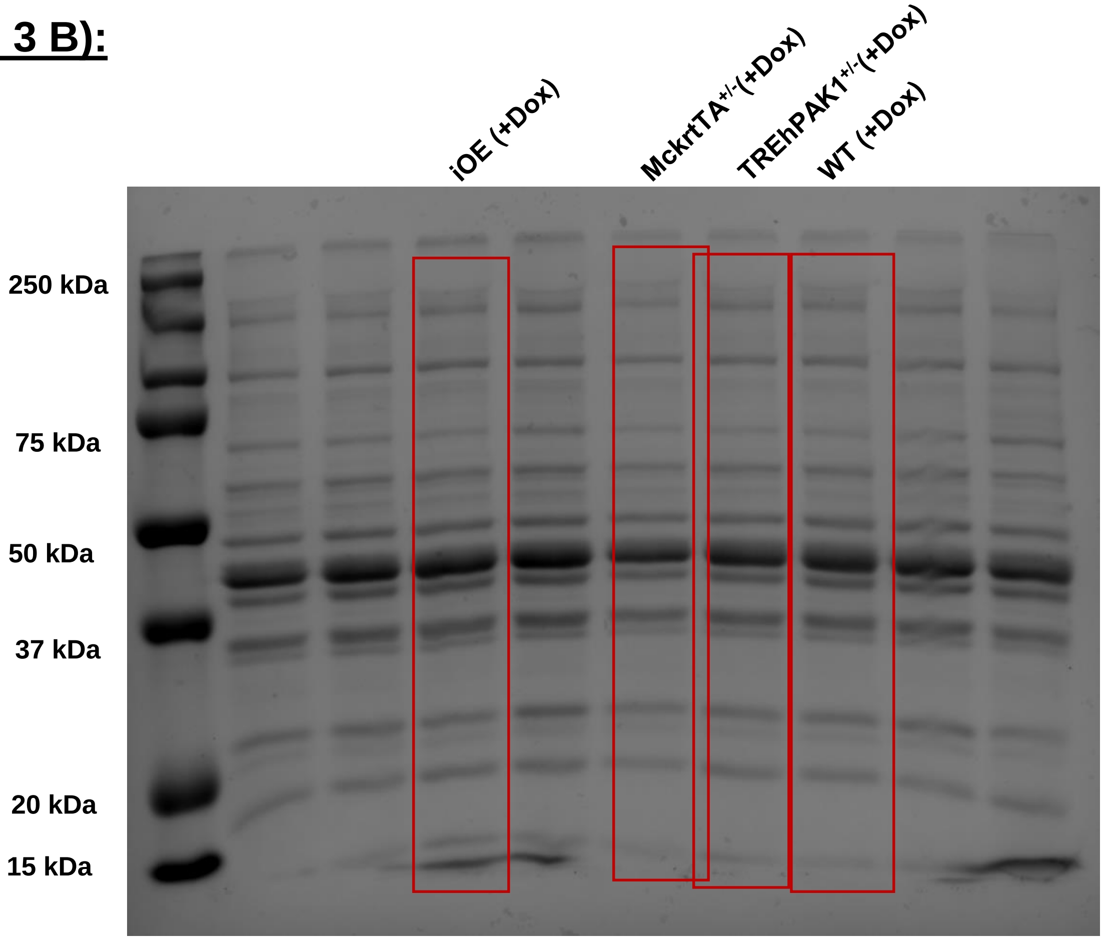

Figure 3 H):

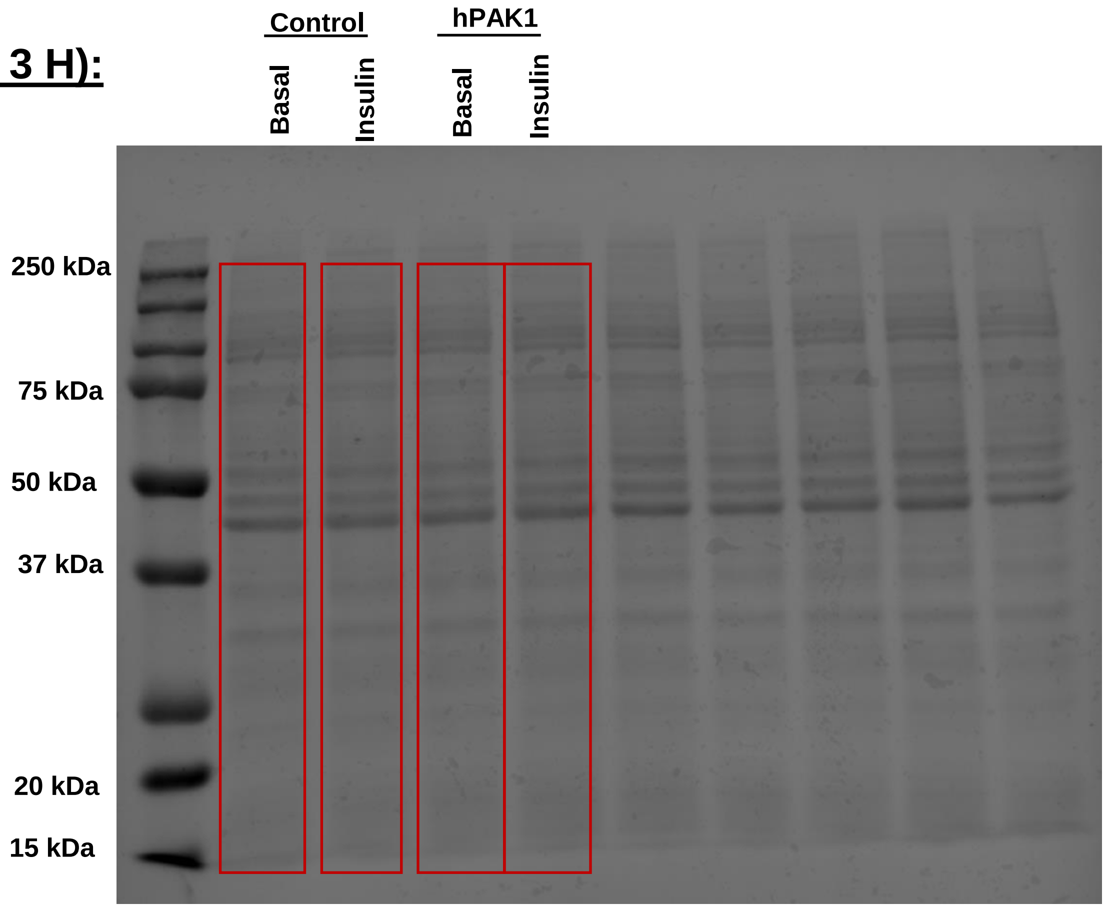

Figure 4 B):

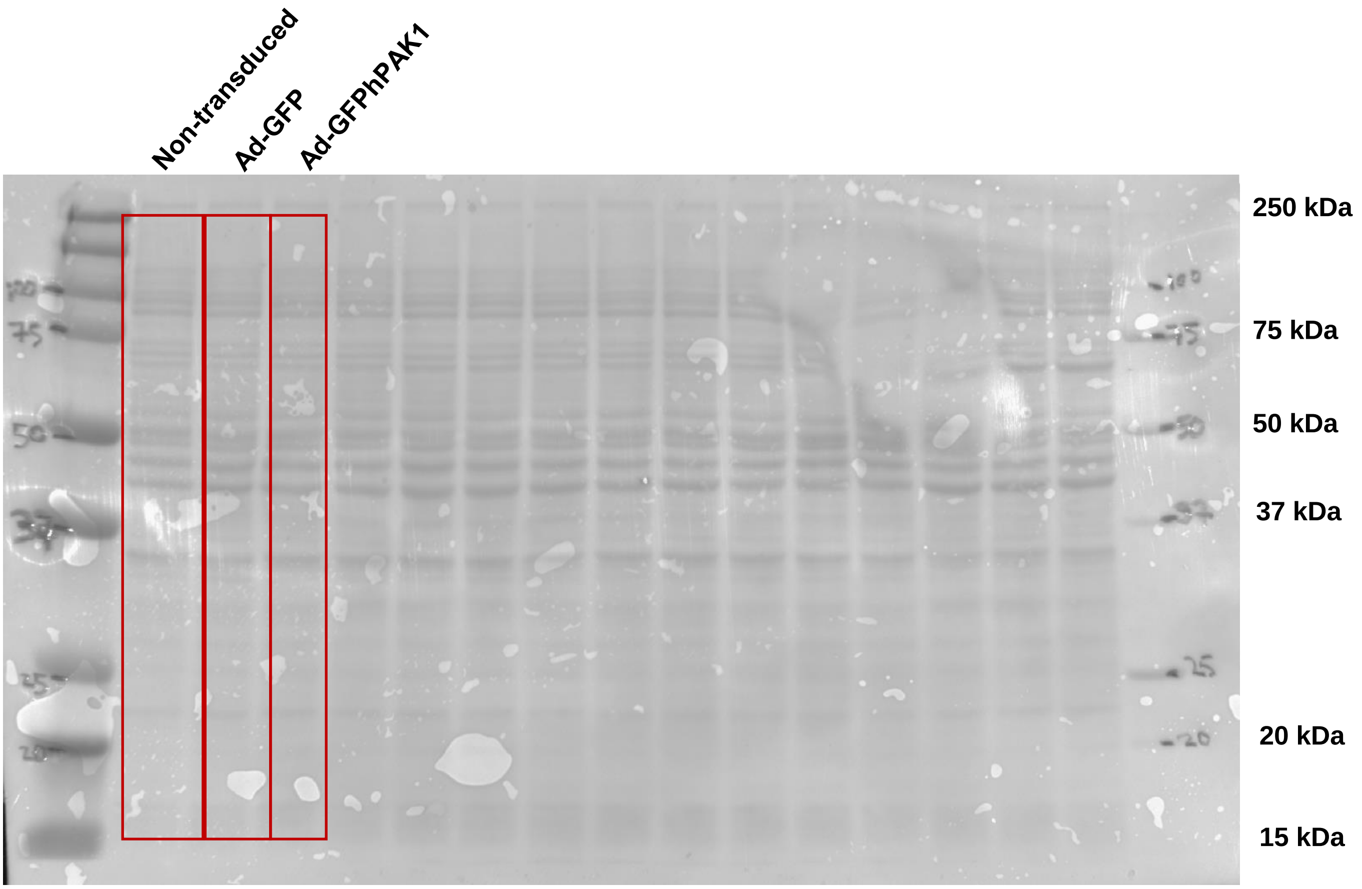
Non-transduced
Ad-GFP
Ad-GFPhPAK1
250 kDa
75 kDa
50 kDa
37 kDa
20 kDa
15 kDa

Figure 5 B):

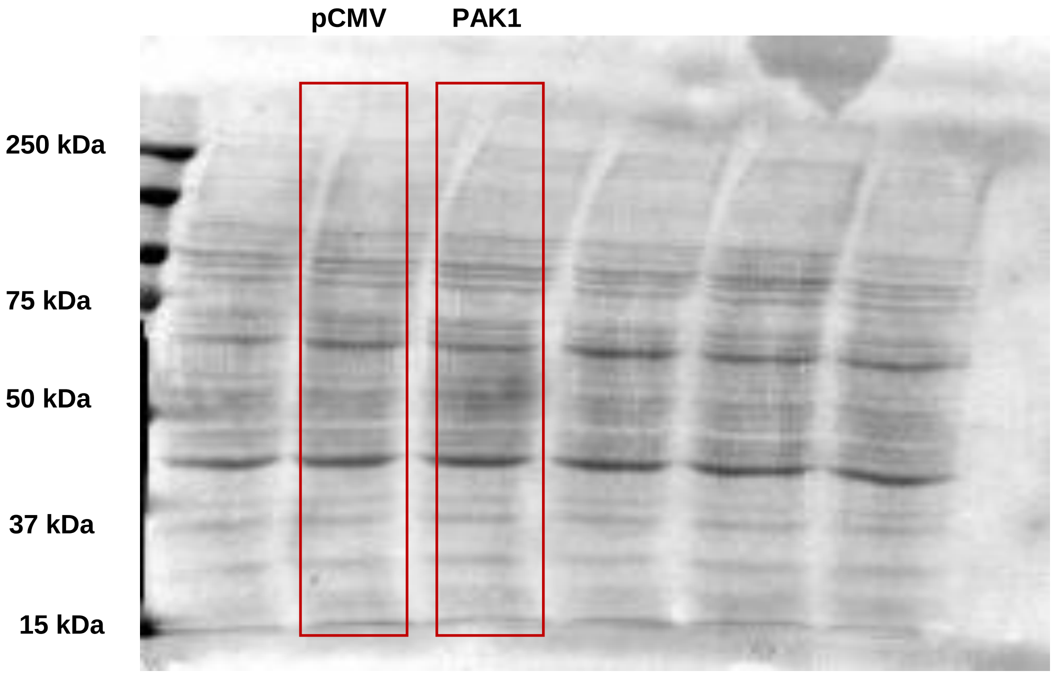

Supplemental Figure 2 B):

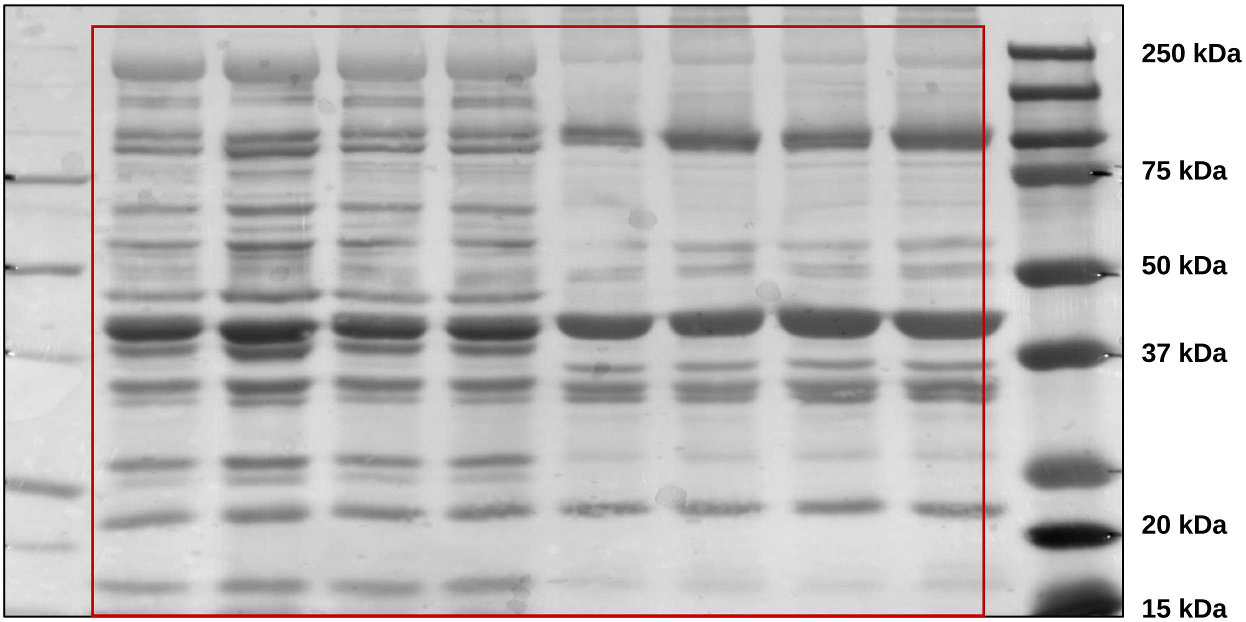

Supplemental Figure 2 C):

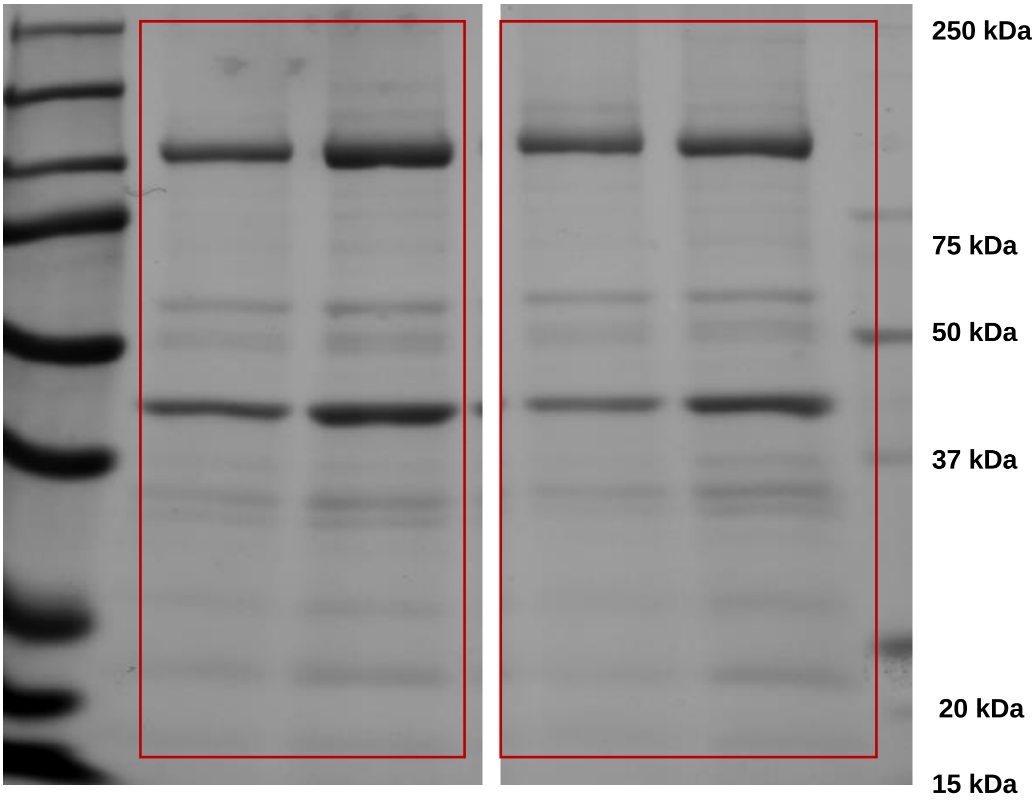

Supplemental Figure 3 A):

Skeletal muscle-PAK1

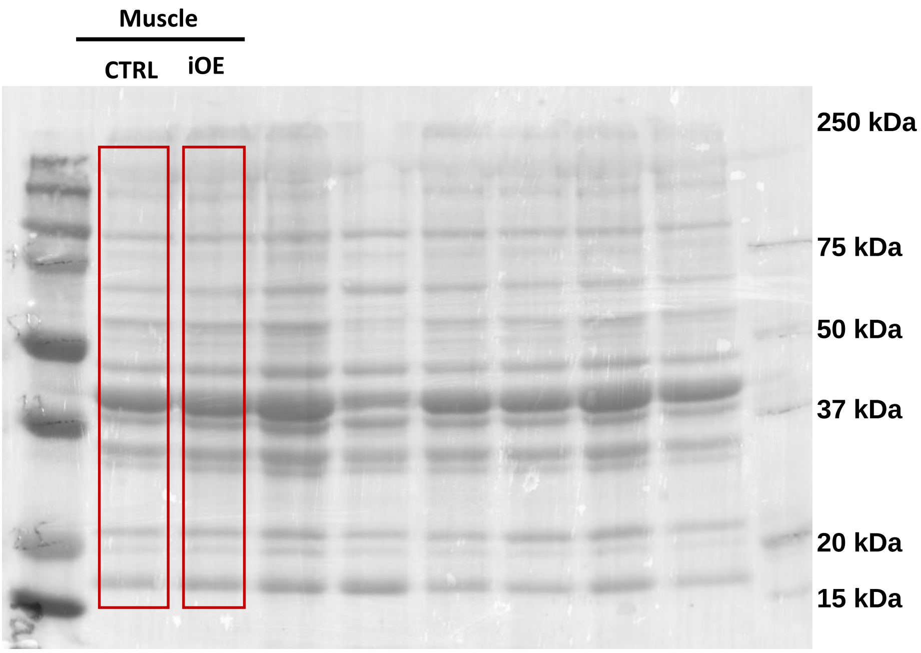

Skeletal muscle-GLUT4

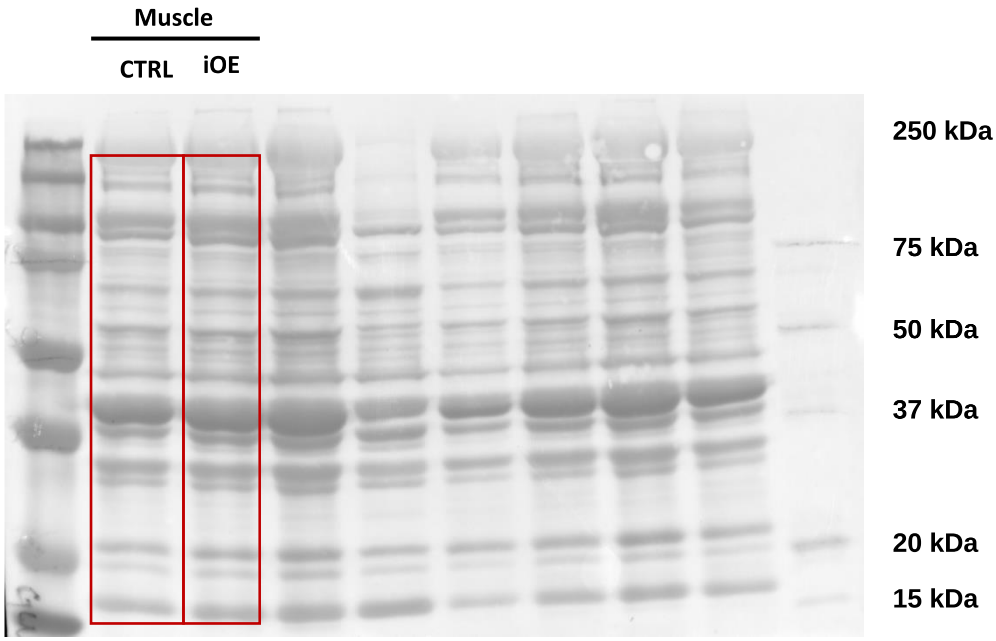

Supplemental Figure 3 A):

Heart-PAK1

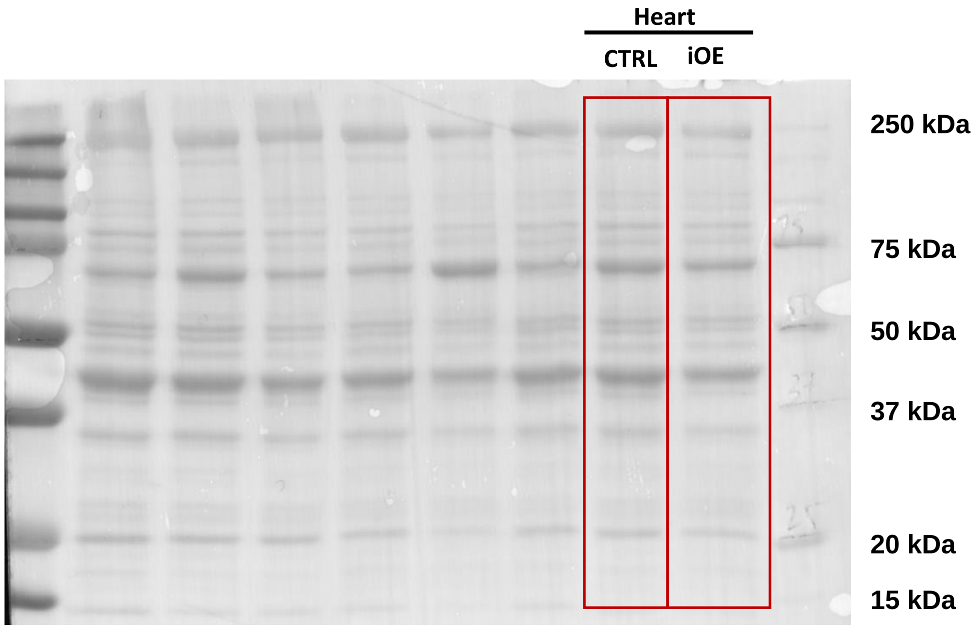

Heart-GLUT4

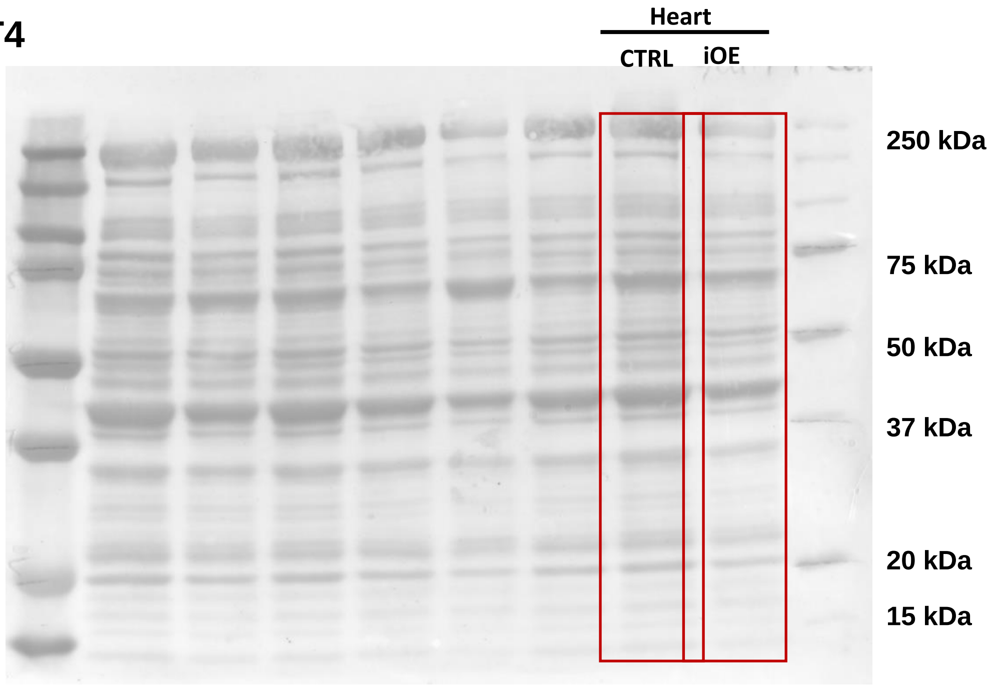

Supplemental Figure 3 A):

Liver-PAK1

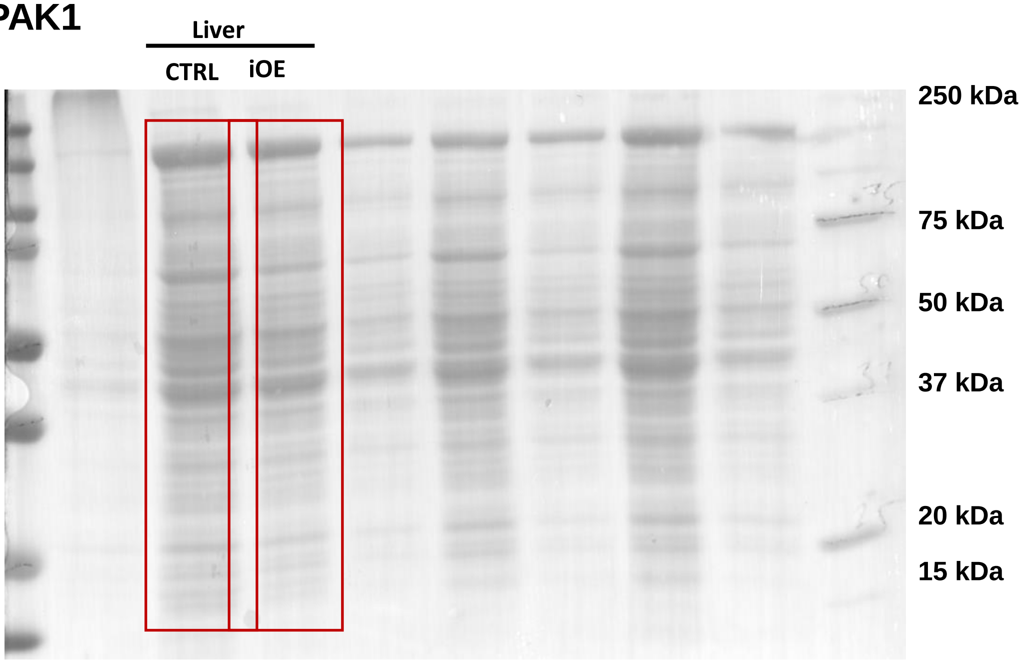

Liver-GLUT4

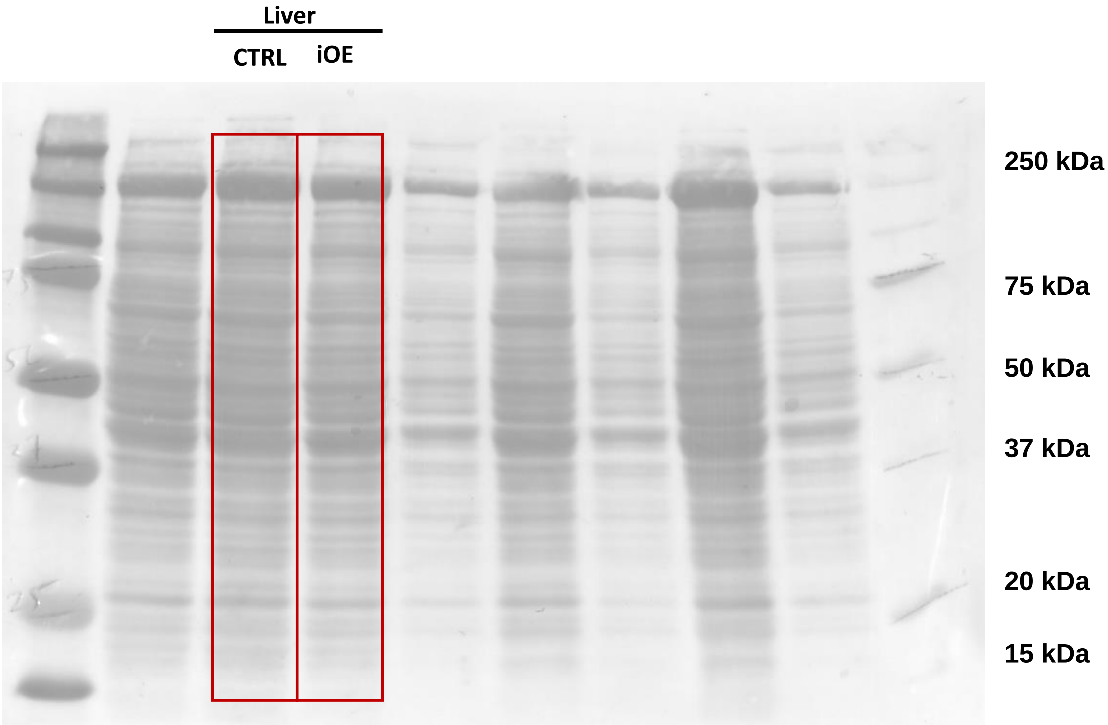

Supplemental Figure 3 B):

Skeletal muscle

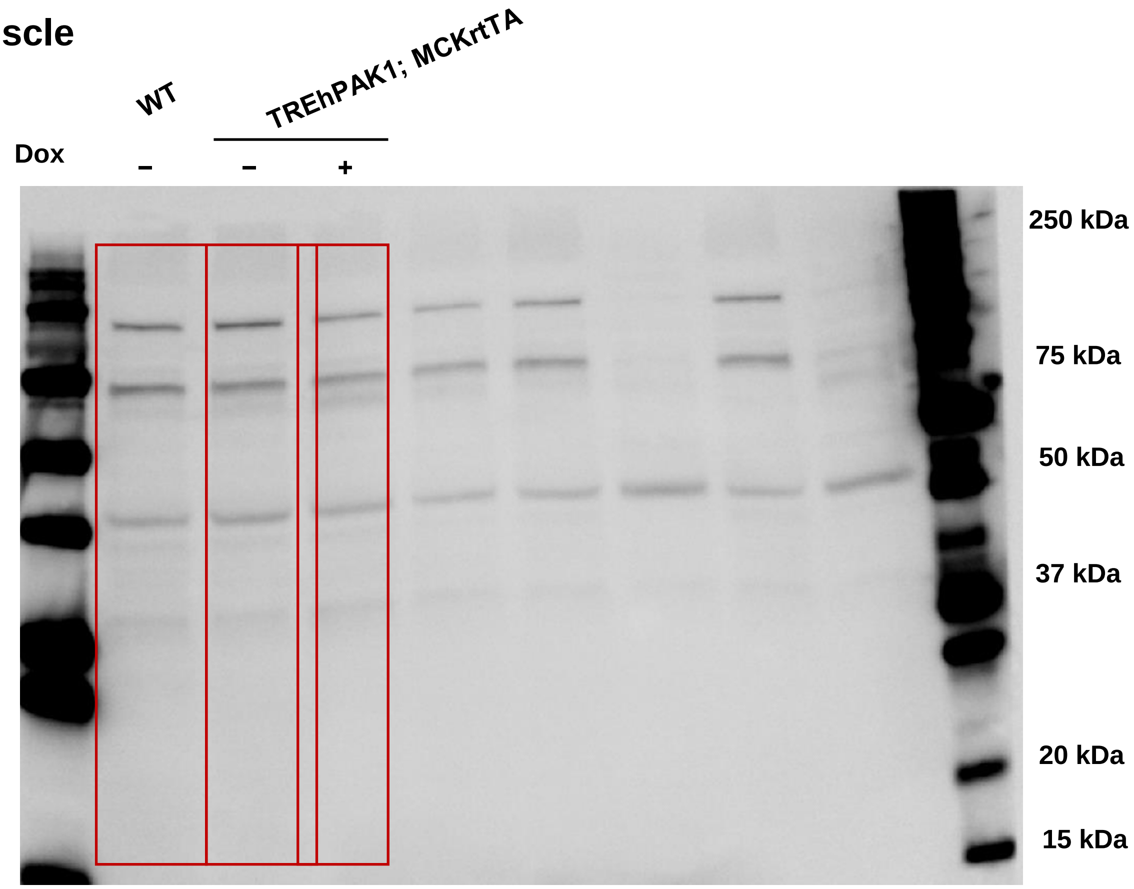

Supplement: Supplementary file 2 [file DataSheet_2.pdf]
